# Spatial Architecture of GLUT1 and Glycolytic Complexes in Breast Cancer

**DOI:** 10.3390/cells15191726

**Published:** 2026-09-22

**Authors:** Luca Secco, Martina Mascaro, Michele Costanzo, Lucia Santorelli, Riccardo Sgarra, Sara Petrosino

**Affiliations:** 1 Department of Life Sciences, University of Trieste, 34127 Trieste, Italy; luca.secco@phd.units.it; 2 Department of Medicine, Università degli Studi di Udine, 33100 Udine, Italy; martina.mascaro@uniud.it; 3 Department of Molecular Medicine and Medical Biotechnology, University of Naples Federico II, 80131 Naples, Italy; michele.costanzo@unina.it; 4 Center for Genetic Engineering (CEINGE)-Biotecnologie Avanzate Franco Salvatore, 80145 Naples, Italy; 5 Clinical Proteomics and Metabolomics Unit, School of Medicine and Surgery, University of Milano-Bicocca, Vedano al Lambro, 20900 Monza, Italy; l.santorelli@campus.unimib.it; 6 Department of Basic Biotechnological Sciences, Intensive Care and Perioperative Clinics, Catholic University of the Sacred Heart, 00168 Rome, Italy

**Keywords:** breast cancer, GLUT1, glycolytic metabolon, membrane scaffolding, biomolecular condensates, protein–protein interactions, liquid–liquid phase separation, XL-MS

## Abstract

Breast cancer cells exhibit a tight dependence on glucose metabolism, sustained both by the transcriptional deregulation of metabolism-related genes and the spatiotemporal organization of the glycolytic machinery across cellular compartments. Although the overexpression of GLUT1 and key glycolytic enzymes is a well-established hallmark of aggressive breast cancer subtypes, expression-level analyses sometimes fail to predict the increased glycolytic flux in breast cancer cells. Subcellular localisation is in fact a critical, yet underappreciated, regulatory layer controlling glycolytic flux. Key mechanisms contributing to this include the recruitment of glycolytic enzymes into dynamic cytoplasmic assemblies, the association of glycolytic enzymes and GLUT1 with membrane scaffolding platforms that concentrate glycolytic activity at sites of glucose entry and utilization, and their co-recruitment to endocytic vesicles enabling compartment-specific metabolic support. Specific protein–protein interactions determine these scenarios. We review both the conventional and emerging techniques to resolve the organizational complexity, as well as the functional and clinical implications, of this spatial regulatory layer in breast cancer. We highlight the spatial organization of GLUT1 and glycolytic enzymes as a critical determinant of glycolytic flux and suggest that disrupting their architecture may lead to new therapeutic strategies in breast cancer.

## 1. Introduction

Breast cancer (BC) cells reprogram their metabolism to sustain energetic and bio-synthetic needs, and the acquisition of aerobic glycolysis—the conversion of glucose to lactate in the presence of oxygen—represents one of the most notable and clinically relevant adaptations [1,2]. This phenomenon, known as the Warburg effect, is characteristic of the most aggressive BC subtypes [3]. BC is notable for its heterogeneity [4] and is classified into five intrinsic subtypes: luminal A, luminal B, HER2-overexpressing, basal-like, and normal-like tumours [5]. The basal-like subtype, which has a high grade of aggressiveness and a high proliferation rate, is commonly referred to as triple-negative breast cancer (TNBC) because of its negative immunophenotype for estrogen receptor (ER), progesterone receptor (PR), and human epidermal growth factor receptor 2 (HER2) [6]. TNBC exhibits the highest glycolytic activity compared with luminal BC, as evidenced by significantly elevated ^18^F-FDG uptake [7]. Aerobic glycolysis plays a pivotal role in TNBC by promoting oncogenic signaling, proliferation, and a metastatic phenotype [8].

Glucose transporters (GLUTs) are integral membrane transporters that mediate glucose uptake and are encoded by the *Solute Carrier Family 2* (*SLC2A*) gene family. GLUT1, encoded by the *SLC2A1* gene, plays a central role because of its ubiquitous expression and is responsible for basal glucose uptake in many tissues [9]. Once inside the cell, glucose can enter several metabolic pathways, including glycolysis, which converts glucose into pyruvate. Pyruvate can then be processed anaerobically into lactate or, when oxygen is available, can enter the Krebs cycle [10].

Beyond enzyme concentration and well-known classical regulatory mechanisms, glycolytic flux also depends on the organization of GLUT1 and glycolytic complexes, which is sustained by transient and dynamic protein–protein interactions (PPIs) [11,12]. Classical biochemical approaches are often insufficient to capture these interactions, which may help to decipher the spatial relationship between glycolytic enzymes (GEs) and GLUT1 across distinct subcellular compartments and represent an underappreciated regulatory determinant of glycolytic flux in BC.

This review aims to integrate information on how cancer cells control glycolytic flux, focusing in particular on the supramolecular layer of modulation involving the spatiotemporal organization of transporter and enzyme complexes. The functional and clinical implications of this spatial regulatory layer are discussed, including its contributions to therapeutic resistance and the potential to exploit the spatial co-organization of GLUT1 and glycolytic complexes as a vulnerability in BC treatment.

We also reviewed the main biochemical and proteomic approaches available for studying PPIs, including cross-linking mass spectrometry (XL-MS), which remains one of the most powerful strategies for resolving the organizational complexity of PPIs within both membrane-defined compartments [13] and membrane-less condensates [14].

## 2. GLUT1 Transcriptional Program and Signaling Networks in Breast Cancer

Increased glucose transport in BC cells is commonly associated with elevated and deregulated expression of GLUTs, including GLUT1. GLUT1 is the best characterized GLUT in BC, and its expression is significantly associated with high histological grade, basal-like phenotype, increased proliferative index, and poor clinical outcome, supporting its role as a prognostic biomarker of aggressive disease [15,16,17]. Higher expression of GLUT1 in tumours compared with normal tissues is orchestrated at both transcriptional and post-transcriptional levels [18,19]. In particular, transcriptional regulation of GLUT1 in BC is driven by increased activity of activating transcription factors (TFs) and loss of repressive TFs.

Among these, epidermal growth factor receptor (EGFR) activation enhances glucose uptake through multiple converging mechanisms, primarily involving the PI3K/Akt signaling pathway. One well-characterized mechanism involves thioredoxin-interacting protein (TXNIP), a negative regulator of GLUT1 abundance at the plasma membrane (PM) [20]. Akt-mediated phosphorylation of TXNIP disrupts its interaction with GLUT1, increasing GLUT1 membrane localization and consequently glucose uptake [21]. In addition, EGFR signaling suppresses TXNIP expression, further facilitating glucose uptake. Akt activity is further supported by mechanistic target of rapamycin complex 2 (mTORC2), which phosphorylates Akt at Ser473, while EGFR also stimulates the transcription factor c-Myc, leading to increased GLUT1 expression and reduced TXNIP transcription. Collectively, these mechanisms enhance glucose uptake and support the increased metabolic demands of proliferating cells [21].

c-Jun may represent a key TF through which GLUT1 promotes BC progression via metabolic reprogramming. Indeed, it has been demonstrated that the GLUT1/c-Jun axis contributes to BC metastasis, highlighting this signalling pathway as a potential therapeutic target [22].

Hypoxia-Inducible Factor 1-alpha (HIF-1α) binds functional Hypoxia Response Elements (HREs) in the *SLC2A1* promoter region to induce GLUT1 mRNA expression under hypoxia [23], a condition particularly relevant in cancer, including BC [24]. Similarly, c-Myc induces *SLC2A1* transcription [25], and its amplification drives glycolytic reprogramming and poor prognosis in BC [26].

Conversely, the WW domain-containing oxidoreductase (WWOX) tumour suppressor has been identified as a physical and functional interactor of HIF-1α, thereby regulating its transactivation function [27]. While loss of WWOX results in activation of glycolysis, genetic or pharmacological reduction in HIF-1α rescues WWOX-regulated phenotypes associated with its depletion, including tumorigenesis. Moreover, BC samples exhibited an inverse correlation between WWOX and GLUT1 expression, a direct HIF-1α target, indicating that WWOX suppresses GLUT1 expression and, consequently, glucose uptake and glycolytic metabolism [27,28]. Further, wild-type p53 represses GLUT1 transcription through direct promoter binding, an effect lost upon *TP53* mutation, which is associated with broad upregulation of glycolytic gene expression across 762 TCGA BC samples [29,30].

In light of this, GLUT1 is a key determinant of enhanced glucose uptake in BC, particularly in aggressive TNBC [31]. Functional studies demonstrate that GLUT1 is required during the early stages of mammary tumorigenesis in vitro and in vivo [32]. Although GLUT1 has traditionally been regarded as a passive facilitator of glucose uptake, accumulating evidence indicates that it actively participates in the organization of signaling networks governing tumour progression [17]. GLUT1 dynamically integrates extracellular nutrient availability with intracellular signaling pathways, thereby influencing proliferation, migration, epithelial-to-mesenchymal transition (EMT), metabolic reprogramming, and therapeutic resistance [33]. Molecular analyses further revealed that GLUT1 regulates both the EGFR/MAPK and integrin β1/Src/FAK signaling cascades, identifying GLUT1 as an upstream modulator of receptor-mediated oncogenic signaling rather than merely a passive metabolic transporter [34]. Indeed, it has been demonstrated that GLUT1 is required in the early stages of mammary tumorigenesis in vitro and in vivo [32]. Similarly, transcriptional regulators extend their control beyond GLUT1 to coordinate the expression of key GEs.

## 3. Glycolytic Enzyme Transcriptional Program and Moonlighting Function in Breast Cancer

The deregulation of GEs has been extensively documented in BC. Hexokinase 2 (HK2) and phosphofructokinase-1 (PFK1), two of the three rate-limiting enzymes of glycolysis, are overexpressed across BC subtypes, with HK2 levels correlating with advanced clinical stage, nodal metastasis, and poor patient survival [35,36,37].

HIF-1α is established as a core transcriptional regulator of glycolytic reprogramming in cancer, activating the expression of genes encoding glucose transporters (i.e., GLUT1) and most GEs through binding to HREs [38]. This is supported by evidence showing that Nuclear factor erythroid 2-related factor 2 (NRF2) sustains HIF-1α activity to drive HK2-dependent glycolysis and tumour growth [39]. Among PFK1 isoforms, expression of the platelet-type isoform of PFK (PFKP) is positively associated with receptor-negative status and reduced overall survival (OS) in BC patients [40]. Furthermore, expression of the liver-type isoform of PFK (PFKL) correlates with glycolytic metabolism and aggressiveness across BC cell lines [41]. At the transcriptional level, direct regulatory mechanisms controlling PFKL expression in BC remain largely uncharacterized. By contrast, PFKP transcription is directly regulated by at least three factors with demonstrated promoter occupancy: activation by Krüppel-like factor 4 (KLF4), and repression by both the BRCA1–ZBRK1 co-repressor complex and SNAI1/Snail factors [37,42,43].

In addition, Myc directly induces HK2 transcription, thereby enhancing aerobic glycolysis and promoting tumorigenesis [44,45]. Tumour necrosis factor-α (TNFα) released by tumour-associated macrophages activates IKK-mediated Yes-associated protein 1 (YAP) phosphorylation, which in turn cooperates with NF-κB/p65 to synergistically drive HK2 transcription, thereby enhancing aerobic glycolysis and promoting cell migration [46]. Converging evidence indicates that Signal Transducer and Activator of Transcription 3 (STAT3), activated by distinct upstream signals, consistently upregulates HK2 transcription in BC, thereby enhancing aerobic glycolysis and contributing to tumour progression, cancer stem cell maintenance, and therapeutic resistance [47,48,49].

Current evidence indicates that Pyruvate kinase M2 (PKM2) and HK2 have a verified moonlighting role as direct regulators of gene expression and as structural scaffolds in BC [50,51]. PKM2 acts as a direct transcriptional co-activator, binding HIF-1α and recruiting p300 while phosphorylating histone H3 at Thr11 to open chromatin at HRE, thereby driving expression of glycolytic genes including lactate dehydrogenase A (LDHA) and GLUT1; it also phosphorylates STAT3 at Tyr705, establishing a self-amplifying STAT3–PKM2–HIF-1α loop documented in TNBC cells. Evidence shows that PKM2 dictates the poised chromatin state of the 6-phosphofructo-2-kinase/fructose-2,6-bisphosphatase 3 (PFKFB3) promoter and shapes the HIF-1α-driven hypoxic transcriptome in luminal A BC cells, directly linking nuclear PKM2 to BC progression [50].

Further, HK2 forms a non-catalytic scaffolding complex with Glycogen synthase kinase 3 beta (GSK3β) and protein kinase cAMP-dependent type I regulatory subunit alpha (PRKAR1a) that drives EMT and metastasis, independent of its catalytic glycolytic activity [51].

These observations support the idea that the overexpression of GEs together with GLUT1 constitutes the molecular basis of the Warburg effect in BC, leading to their adoption as prognostic biomarkers and therapeutic targets [52].

## 4. GLUT1 and the Glycolytic Machinery as Biomolecular Condensate

The *SLC2A* gene family encodes 14 distinct isoforms with conserved structural features: they are integral membrane transporters that span the PM bilayer twelve times. Among the 14 GLUTs, GLUT1 is ubiquitously expressed; depending on tissue specificity, its expression occurs together with that of other members of the *SLC2A* gene family. GLUT1 is primarily responsible for glucose transport in glial cells, endothelial cells (ECs), and erythrocytes, which are highly dependent on glucose supply. In the liver, it is expressed in conjunction with *SLC2A2*/GLUT2 [9], while *SLC2A3*/GLUT3 mediates high-affinity glucose uptake in neurons. *SLC2A4*/GLUT4 mediates insulin-dependent glucose transport in muscle and adipose tissues [53]. With few exceptions [54], GLUT1 is insulin-insensitive and does not respond to insulin signaling [55]. Once inside the cell, glucose enters the glycolytic pathway, a series of enzymatic steps that culminate in pyruvate synthesis, accompanied by ATP and NADH production [10].

Glycolysis is the gating pathway for ATP synthesis, and several well-known regulatory mechanisms have evolved to control glycolytic flux according to cellular metabolic requirements. Alongside classical, well-established enzyme-centred mechanisms based on allosteric modulation and post-translational modifications (PTMs), alternative flux modulatory strategies are emerging.

Increasing evidence indicates that many types of cellular structures lacking surrounding membranes can maintain high levels of molecular organization and specific functions; these include P-bodies, stress granules, the nucleolus, Cajal bodies, nuclear speckles, and promyelocytic leukaemia bodies (PMLBs). Collectively referred to as membrane-less organelles (MLOs) or biomolecular condensates, these structures are formed by the coordinated assembly of proteins and/or nucleic acids into highly dynamic compartments [56].

Liquid–Liquid Phase Separation (LLPS) is emerging as a primary mechanism by which macromolecules self-assemble into a concentrated phase when the attractive forces between molecules exceed the entropic cost associated with demixing. LLPS provides an effective means of compartmentalising cellular functions while allowing for rapid molecular exchange [57,58].

### 4.1. From Liquid–Liquid Phase Separation to Metabolic Assemblies

LLPS occurs when macromolecules in solution, such as proteins or RNA, demix and form a two-phase system composed of a dense phase and a dilute, or bulk, phase [57]. This phenomenon is governed by fundamental thermodynamic principles, specifically free energy and chemical potential [57]. The Gibbs free energy (G) is a thermodynamic function used to determine whether a reaction is spontaneous, and it depends on enthalpy (H), temperature (T), and entropy (S). In homogeneous solutions, S favours the random distribution of molecules throughout the available volume (Figure 1A).

However, when attractive intermolecular interactions become sufficiently favourable, the resulting reduction in G allows for spontaneous demixing into coexisting dense and dilute phases under appropriate physiological conditions. This transition occurs only when the molecular concentration exceeds a critical saturation threshold (Csat), above which condensation minimises the overall G of the system [58] (Figure 1B).

Within the cellular microenvironment, condensate formation represents a reversible equilibrium process rather than irreversible aggregation, allowing for rapid adaptation to changing cellular conditions [57,58,59]. LLPS is therefore intimately linked to variations in protein abundance, which are governed by both transcriptional and post-transcriptional events, including PTMs and degradative pathways.

### 4.2. Multivalency and Flexibility

LLPS droplets are enriched in multivalent molecules [60]. Multivalency, one of the fundamental requirements for phase separation (PS), is the ability of molecules to establish multiple simultaneous interactions. Distinct elements govern intra- and intermolecular interactions, such as modular domains or low-complexity amino acid sequences with repeated interaction motifs [58]. When multivalent elements interact (Figure 1B), they assemble into large polymers, decreasing the solubility of those molecules and further promoting their PS [61]. This spatial proximity of sequential enzymes provides the structural basis for substrate channelling: metabolic intermediates are transferred directly between adjacent active sites without diffusing into the bulk cytosol, increasing local reaction efficiency (Figure 1C). Experimental studies have demonstrated that increasing the number of interaction sites substantially lowers the concentration threshold required for condensate formation: Csat decreases dramatically with an increase in valency, allowing for droplet formation at lower molecular concentrations [60,62]. Conversely, affinity does not follow the same trend as valency: high-affinity monovalent molecules have been shown to block LLPS. This occurs because high affinity decreases both the dynamicity of the droplet and its rapid exchange with the surrounding medium, which are two fundamental properties of liquid droplets [60,63]. LLPS is sustained by cooperative networks of individually weak interactions (Figure 1B). Electrostatic interactions arise between oppositely charged residues and strongly depend on charge distribution rather than overall amino acid composition [64]. Among the weak molecular interactions relevant to LLPS are: hydrogen bonds, important in protein folding; hydrophobic interactions, contributing to the initiation and reinforcement of LLPS [65]; Van der Waals forces, among which are stronger dipole–dipole forces and weaker dispersion forces, influencing molecular packing and structural organization [66]; π–π and cation–π interactions, contributing significantly to condensate stability [64,65] (Figure 1B).

### 4.3. Scaffold and Client Components

Biomolecular condensates are composed of two types of molecules: scaffold and client molecules (Figure 1). Scaffolds are the primary drivers of LLPS and are essential for the structural integrity and stability of the droplet [62]. Deletion of these components reduces the size or number of the respective structures [67]. In contrast, clients constitute the remaining majority of molecules; they are not strictly necessary for condensate formation because they cannot undergo PS on their own. Since clients are generally more abundant than scaffolds, scaffolds are typically present at a higher relative density within the droplet network to coordinate client recruitment [67]. In this context, multivalency can be achieved by: (i) folded proteins containing multiple interaction domains that can interact with short linear motifs (SLiMs) of other proteins [57]; (ii) intrinsically disordered regions (IDRs) or intrinsically disordered proteins (IDPs), which can serve as scaffolds for multiple SLiMs [68]; and (iii) RNA and DNA molecules, which can act as robust scaffolds for other nucleic acids or proteins through repeated motifs or specialised secondary structures [58] (Figure 1). IDPs and IDRs lack stable tertiary structures and instead exist as dynamic ensembles of conformations. Their flexibility enables numerous transient interactions that are highly compatible with LLPS [64]. These proteins interact in different ways depending on the specific modules involved, which include molecular recognition features (MoRFs), SLiMs, and low-complexity regions (LCRs) [69,70]. However, disorder alone is not sufficient to induce LLPS, as phase separation also depends on sequence organization, multivalency, and environmental conditions [71]. Proteinaceous molecules are not the only components present in condensates. Nucleic acids, particularly negatively charged RNA molecules, interact electrostatically with positively charged protein domains, promoting LLPS. Further, repeated RNA-binding motifs allow RNA molecules to function as multivalent scaffolds for RNA-binding proteins [72]. Beyond nucleation, RNA influences condensate composition, molecular recruitment, viscosity, and exchange kinetics. Distinct RNA species contribute to the molecular identity of different MLOs, suggesting that nucleic acids play both structural and regulatory roles [62,72].

### 4.4. The Glucosome as a Phase-Separated Condensate: Evidence and Open Criteria

It has been demonstrated that GEs can organise into cytoplasmic multienzymatic aggregates known as glucosomes. Glucosomes are composed of multiple enzymes involved in glucose metabolism, including PFKL, fructose-1,6-bisphosphatase (FBPase), and PKM2. Current evidence suggests that PFKL acts as the core scaffold molecule within the glucosome, forming cytoplasmic clusters and recruiting the GEs into the same assemblies [11]. However, whether the glucosome formation is based on an LLPS process has not yet been clearly elucidated.

Several lines of evidence support a phase separation behavior. First, fluorescence resonance energy transfer (FRET) experiments have demonstrated direct interactions between PFKL and FBPase, supporting the existence of genuine multienzymatic assemblies rather than simple spatial co-localisation [11]. To support this dynamic behavior, fluorescence recovery after photobleaching (FRAP) experiments have demonstrated continuous molecular exchange within PFKL clusters in BC cells. This indicates that these structures remain highly dynamic and reversible (Figure 1C), rather than representing insoluble, pathological aggregates. Furthermore, the diffusion of PFKL inside the condensates is substantially slower than in the surrounding cytoplasm, reflecting a dense network of intermolecular interactions while preserving internal mobility [11]. Moreover, computational modelling further predicts that PFKL filament elongation promotes multivalency and increases cluster frequency and size [73]. Finally, it is noteworthy that GEs, including Pfk2p and PFKL, have been shown to coalesce into MLOs called glycolytic bodies (G bodies) in Saccharomyces cerevisiae and human hepatocarcinoma cells, respectively, under hypoxic stress [74]. In yeast, G bodies have been demonstrated to phase-separate, showing important features, such as multivalent assembly, in vivo fusion, and RNA-dependent formation [75]. However, they are not as sensitive to 1,6-hexanediol, an LLPS-disrupting agent, suggesting a gel-like rather than a purely liquid state [75]. Although some hallmarks of LLPS are present in glucosome formation and organization, fluorescence recovery and punctate localization alone are not sufficient to establish an LLPS mechanism. To our knowledge, several criteria typically used to distinguish bona fide LLPS have not yet been tested for the glucosome, especially sensitivity to 1,6-hexanediol. Local PFKL concentration also emerges from computational modelling as a key parameter driving condensate formation [73], but a concentration-dependent nucleation threshold has not been experimentally measured in cells.

## 5. Conventional and Recent Approaches for Protein–Protein Interaction Analysis

For decades, the identification of PPIs has relied on biochemical and biophysical techniques such as co-immunoprecipitation (Co-IP), affinity purification coupled to mass spectrometry (AP-MS), yeast two-hybrid screening (Y2H), FRET, bioluminescence resonance energy transfer (BRET), proximity ligation assay (PLA), and surface plasmon resonance (SPR). Each methodology provides unique and complementary information regarding protein association, binding affinity, interaction kinetics, or spatial information. A comparative summary of these approaches is provided in Table 1.

The Y2H system was the first widely adopted high-throughput technology for identifying binary PPIs in living cells and played a foundational role in mapping early interactomes [76]. The foundational Y2H study identified GLUT1 C-terminal binding protein (GLUT1CBP/GIPC1) as a direct, isoform-specific GLUT1 C-terminal PDZ-domain partner, linking GLUT1 to the cytoskeleton [77]. Continuous technological developments, including membrane-adapted systems and next-generation sequencing-based variants, have expanded its applicability while improving sensitivity and reducing false positives [78]. Classical biochemical approaches such as co-IP, AP-MS and pull-down strategies represented the first major steps beyond binary assays, allowing for the isolation of native protein complexes directly from their physiological environment [79], and still remain the gold standard for identifying stable protein complexes on a proteome-wide scale [80,81]. An initial Y2H screen followed by Co-IP confirmed that HK2 forms a ternary, non-catalytic scaffolding complex with GSK3β and PRKAR1a, promoting EMT and metastasis [51]. On the other hand, FRET and BRET enable real-time monitoring of molecular proximity in living cells [82,83]. PLA, including recent systems such as TurboID variants and APEX2 constructs, allows for visualization of endogenous interactions within intact cells, tissues and organisms [84], while SPR provides quantitative measurements of binding kinetics of complexes in real time [85]. Despite their broad applicability, these approaches often fail to detect weak, transient, or compartment-specific interactions, particularly those involving membrane proteins or rapidly assembled signaling complexes. Consequently, orthogonal validation combining multiple experimental techniques has become essential for establishing the biological relevance of newly identified PPIs. Because PPIs are often weak and rapidly reversible, conventional purification workflows frequently disrupt the very assemblies they aim to capture in their native, multivalent, spatially constrained environment. This limitation has prompted the integration of strategies capable of preserving native cellular context and capturing transient, polyvalent interactions. In this context, XL-MS combines chemical stabilisation with the high resolution of MS to transiently “freeze” protein interactions prior to extraction, significantly improving the detection of labile complexes [13]. A notable example is the mitochondrial Krebs cycle metabolon, where XL-MS has generated structural evidence for enzyme proximity and substrate channelling, revealing previously unresolved interaction interfaces among Krebs cycle enzymes [86]. XL-MS has been directly applied to MLOs and LLPS systems, and is increasingly regarded as a dedicated methodology. Boczek and colleagues developed a quantitative, time-resolved XL-MS (qXL-MS) approach to monitor PPIs and conformational dynamics inside condensates formed by the RNA-binding protein FUS. Their method allowed condensate-specific interactions to be distinguished from those present in the dispersed phase [87]. Although XL-MS provides residue-level information on protein–protein contacts, it may offer only a partial view of the structural landscape, as it does not capture the broader conformational rearrangements that accompany assembly or phase transitions. Limited proteolysis MS (LiP-MS) fills this gap by detecting proteome-wide structural changes in native conditions, making it particularly suited to the dynamic and partially disordered proteins that populate protein assemblies, metabolons and MLOs [88,89].

As the field evolved, PPI strategies were progressively complemented by multi-dimensional live-cell fluorescence-imaging approaches that integrate spatial, temporal, spectral, and photophysical measurements, including FRET, FRAP, fluorescence-lifetime imaging microscopy (FLIM) or combined strategies such as the FLIM-FRET [90]. These modalities expanded the descriptive power of early imaging studies by providing direct access to the dynamic and biophysical properties of MLOs and other assemblies [91]. Indeed, beyond establishing enzyme co-localization, these approaches combine high-resolution spatial mapping with recovery kinetics and quantitative spatial analyses to capture assembly formation, turnover, and reconfiguration in real time.

More recently, structural biology has entered a transformative phase with the application of cryo-electron microscopy (cryo-EM), which now enables visualization of endogenous protein communities with near-native preservation. Cryo-EM has helped to define the structure of PFKL, its tetramers, and tetramer–tetramer filament interfaces in hepatocellular carcinoma cells. This evidence provides strong structural support for regulated, complex-dependent glycolytic enzyme organization [92]. Furthermore, cryo-EM combined with Artificial Intelligence (AI)-assisted particle classification revealed heterogeneous endogenous assemblies that would have been inaccessible through traditional structural methods, offering a promising framework for resolving PPI heterogeneity at near-atomic resolution, further boosted by integrated tools and machine learning algorithms [93]. This transition is particularly significant because, unlike single, well-defined complexes, large heterogeneous ensembles have historically been incompatible with crystallographic approaches. In addition, structural prediction and computational approaches are rapidly becoming indispensable for PPI discovery and validation. In this field, the application of AlphaFold3 marks a major conceptual leap [94]. AI-driven structural prediction was recently used to infer interaction interfaces and assembly architectures of endogenous metabolons that remain experimentally intractable. Rather than replacing experimental methods, these predictive frameworks are increasingly integrated with proteomics, XL data, and functional assays to generate experimentally testable models of protein complex organization [94]. Collectively, these technological advances illustrate a clear methodological evolution, from binary interaction assays to biochemical purification, functional metabolomics, structural proteomics, cryo-EM, and AI-driven modelling. These provide important methodological proof of principle, highlighting the need for dedicated structural interactomics studies in BC models.

## 6. Approaches for Characterizing Protein Interactions of GLUT1 and Glycolytic Enzymes in Breast Cancer

Despite this emerging systems-level perspective, the specific interactome of GLUT1 and GEs remains largely unexplored with modern structural interactomics approaches (Table 1). In particular, no studies to date have systematically characterized GLUT1 and GE-specific PPIs in BC. Existing interactome data rely largely on indirect methods such as affinity purification or computational inference (see Section 5), leaving unresolved the question of which molecular partners physically associate with GLUT1 or GEs in situ and contribute to their oncogenic functions.

To address this gap, we propose leveraging next-generation structural interactomics, specifically in situ XL-MS and proximity labelling, to map the GLUT1/GE interactome at high resolution within its native membrane environment. As a representative example, Amaral and co-workers [95] recently introduced a FIX-MS workflow that couples formaldehyde-based spatial stabilization with NHS-driven lysine labelling, yielding abundant, structurally informative crosslinks without disrupting cellular ultrastructure. This approach is particularly suited to membrane proteins such as GLUTs, whose topology and trafficking dynamics have long limited the effectiveness of classical interactome methods. Notably, in the context of BC, a recent XL-MS dataset generated from patient-derived xenograft models already provides a rich structural interaction landscape [96]. This resource offers an excellent starting point for interrogating the role of GLUT1 and GEs within subtype-specific PPI networks and for positioning their interactome within disease-relevant molecular architectures. Finally, the recently established thermal proteome profiling (TPP) workflow for identifying effector drug–protein interactions in cancer cell lysates provides a robust methodological framework for probing membrane-associated targets [97]. By extending thermal profiling beyond PPI mapping to small-molecule engagement, this streamlined approach offers a practical and robust framework for probing membrane-associated targets. Applied to the GLUT family or GEs, it could enable systematic screening for pharmacologically relevant modulators capable of altering their stability or interaction landscape within disease-relevant cellular environments.

In conclusion, by integrating click-linking or complementary proximity labelling platforms (BioID, TurboID, APEX2), future studies could generate the first comprehensive, structure-aware map of GLUT1 and GE interactions in BC, revealing direct binding partners, regulatory complexes, and actionable molecular nodes previously inaccessible to conventional biochemical approaches.

## 7. GLUT1 Subcellular Localization as a Regulatory Variable

Transcriptional upregulation driven by oncogenic pathways such as HIF-1α or c-Myc sustains GLUT1 expression and may itself promote its PM redistribution [25,38]. However, GLUT1 levels do not universally correlate with increased glycolytic flux, as evidenced by the absence of a significant association between GLUT1 immunoreactivity and ^18^F-FDG uptake in primary BCs [98], and by the finding that high GLUT1 protein levels do not necessarily result in increased glucose transport activity in cancer cell lines [99]. Therefore, both expression levels and subcellular localization should be taken into account; they represent distinct but mutually influential regulatory dimensions of GLUT1 function.

### 7.1. GLUT1 Surface Localization in Breast Cancer

GLUT1 protein abundance is not solely determined by its transcriptional output but is actively shaped by its subcellular localization. This view is supported by evidence that oncogenic pathways sustain GLUT1 accumulation at the PM through a mechanism that prevents GLUT1 endocytosis/lysosomal degradation, stabilising it at the cell surface in BC cells [100,101] (Table 2). Among others, the USP6 N-terminal-like protein (USP6NL)/AKT pathway promotes the stabilization of GLUT1 at the PM, driving a rewiring of BC cell metabolism towards glycolytic fuel [102]. Furthermore, the downregulation of TXNIP plays a relevant role in both GLUT1 expression and localization. Regarding the latter, TXNIP physically interacts with GLUT1, facilitating its recruitment into lysosomal vesicles for degradation. Therefore, TXNIP repression is critical in BC cells [100,101]. This bidirectional relationship places subcellular localization at the centre of the regulatory hierarchy of GLUT1-mediated glucose uptake in BC.

The subcellular localisation of GLUT1 is dynamically regulated by specific PTMs, namely palmitoylation and phosphorylation [109,110,111]. Some of these modifications are directly driven by oncogenic signaling pathways activated in BC. Two phosphorylation sites on GLUT1 have been directly linked to its PM recruitment: Ser226 and Ser490 [110,111]. In particular, Protein Kinase C (PKC)-dependent phosphorylation of Ser226 is necessary for the rapid increase in glucose uptake and enhanced cell surface localization of GLUT1 [111]. Critically, in tissue samples from patients with recurrent BC, phSer226-GLUT1 is found at the epithelial cell periphery, whereas it is undetectable in patients who do not experience recurrence. This localisation is retained in BC metastases. Collectively, this evidence (Table 3) suggests that phSer226-GLUT1 localisation *per se* can predict BC recurrence and clinical outcome [112] (Figure 2A). Moreover, abnormal activation of the Class I phosphatidylinositol 3-kinase complex I (PI3KC1)-AKT pathway leads to preferential GLUT1 expression at the PM in cancer cells [103]. Further, in invasive ductal BC, ultrastructural immunohistochemistry revealed that most GLUT1 is localized at the PM of tumour cells, a pattern that is very weak or undetectable in normal ductal epithelial breast tissue [113].

### 7.2. GLUT1 Localization Within Endocytic Vesicles: Special Case

Mounting evidence challenges the conventional view that GLUT1 and GEs operate exclusively at the PM and in the bulk cytosol, respectively, and instead shows that both associate dynamically with unconventional subcellular compartments [114,115]. The dynamic association of GEs with transporters creates functional membrane transport metabolons that channel substrates obtained from the extracellular compartment directly into cellular metabolism [116]. Recently, it has been shown that, upon growth factor (GF) stimulation, GLUT1 undergoes co-endocytosis with receptor tyrosine kinases (RTKs) into glucose-loaded endocytic vesicles (EVs). Furthermore, GEs assemble a multi-enzyme complex on the surface of these receptor-containing vesicles to execute glycolytic reactions efficiently and concertedly. GEs co-localizing with the periphery of these vesicles are adjacent to mitochondria (Figure 2B). This emerging mechanism (Table 3) drives glucose uptake without necessarily increasing cell-surface GLUT1, representing a new regulatory layer for glycolytic control [114].

## 8. Glycolytic Enzyme Subcellular Localization as a Regulatory Variable in Breast Cancer

Mechanistically, both the activity and the intracellular distribution of HK and PFK1, rather than their total expression levels, correlate with the aggressiveness and invasiveness of human BC, providing emerging evidence (Table 3) that the subcellular redistribution of GEs is a determinant of glycolytic output in BC [117,118].

### 8.1. Cytoskeletal–Glucosome Assembly

The subcellular redistribution of PFK1 toward an actin-enriched membrane fraction has been directly implicated in the enhanced glycolytic flux of BC tissue [119]. Consistent with this, stimulation with clotrimazole, which induces the detachment of GEs (PFK1 and Aldolase) from the cytoskeleton, results in reduced glycolytic flux and consequently the loss of protrusions responsible for BC cell migration [118]. Furthermore, GEs self-organize into newly discovered dynamic F-actin glycolytic waves, self-organizing dynamic patterns of energy-producing GEs, at the cell edge. Alteration of these glycolytic waves changes glycolytic activity and cell migration ability, demonstrating a level of glycolytic flux regulation that supports migrating BC cells [120]. Taken together, this evidence is particularly relevant because the Ras/PI3K/ERK oncogenic pathway enhances dynamic GEs F-actin wave activity in TNBC cells, triggers de novo actin glycolytic wave activity in mammary epithelial cells, and increases invasive behavior across metastatic BC cell lines [121] (Figure 2C). Collectively, the relocalization of GEs from the cytosol to the PM appears to enhance the migratory capacity of BC cells (Table 3).

### 8.2. Cytosolic–Glucosome Assembly

In human TNBC cells, PFKL forms cytoplasmic clusters of variable size with other rate-limiting GEs [11,122] and co-clusters with HK2 and PKM2 at the lamellipodia of migratory TNBC cells, where its specific subcellular localisation is required for migration [123] (Figure 2D). Microscopy studies have identified glucosomes of various sizes, commonly classified as small, medium, and large assemblies. These distinct sizes have been correlated for the first time with specific glucose metabolic pathways and phases of the cell cycle. Small glucosomes (<0.1 µm) are primarily involved in glycolysis; medium-sized assemblies (0.1 µm < x < 3 µm) promote the pentose phosphate pathway (PPP); and larger assemblies (3 µm < x < 8 µm) divert glucose flux into serine biosynthesis. Notably, these large glucosomes are absent in the non-tumour cells used in control studies, suggesting that this particular macromolecular metabolon plays a fundamental role specifically in cancer metabolism, including BC cells (Figure 2D) [11]. Furthermore, BC cells exploit these size-dependent functions to dynamically regulate metabolism throughout the cell cycle: during the G1 phase, smaller glucosomes are more abundant than in the S phase, whereas their number decreases during the G2/M phase. The G1 phase is also characterized by a higher abundance of medium-sized glucosomes, promoting PPP metabolism, while no significant difference in the number of these condensates was detected in G2/M. Conversely, the number of large glucosomes remains constant across all cell cycle phases (Figure 2D) [124]. Collectively, these findings (Table 3) represent emerging, not yet fully consolidated, evidence that glucosome assembly correlates with cell metabolic state and cell cycle in BC cells.

### 8.3. Glucosome Regulatory Mechanisms in Breast Cancer

Physical properties, pH, and PTMs can modulate glucosome interactions by altering residue charge states: phosphorylation can either promote or inhibit condensation depending on the molecular context, acetylation modifies electrostatic properties by neutralizing lysine residues, and methylation and ubiquitination modulate interaction networks and phase behavior [65,71]. The clearest experimentally demonstrated example of this regulation within glucosomes is the acetylation of Lys689 in PFKL. Mutations that prevent acetylation completely abolish PFKL cluster formation, whereas an acetylation-mimicking substitution restores assembly, demonstrating that this PTM is strictly required for glucosome formation in BC cells [11]. Computational modelling further proposes that such modifications increase the effective PFKL–PFKL interaction strength, thereby facilitating condensate nucleation [73].

Glucosome architecture can also be regulated by extrinsic signaling pathways independently of de novo protein synthesis. For instance, activation of the EGF–ERK1/2 signaling cascade promotes the formation of large glucosomes, whereas ERK inhibition shifts the population toward medium-sized structures without substantially altering the total expression levels of the constituent enzymes. These observations suggest that oncogenic signalling controls spatial reorganization of glucosomes rather than protein abundance in BC cells [125]. Moreover, AURKA can indirectly regulate glucosome assembly by modulating downstream metabolites. Because AURKA is known to promote glycolysis and lactate production, its inhibition has been demonstrated to promote the assembly of medium and large glucosomes, effectively shifting glucose metabolism from glycolysis to the PPP or serine biosynthesis in cancer cells [125].

The cell cycle phase itself functions as a regulatory axis controlling glucosome assembly/disassembly, distinct from biochemical mechanisms (PTM, signaling cascades) discussed. Pharmacological arrest at G1 (lovastatin) or G2/M (colchicine), acting through additional mechanisms (HMG-CoA reductase inhibition vs. microtubule disruption), converges on the same phenotype: a marked reduction in large-sized glucosomes in favour of small/medium populations in BC cells [124]. Taken together, these findings suggest that the cell cycle is an upstream, integrative regulatory layer rather than a mere downstream consequence of glucosome dynamics.

## 9. Targeting Approaches for GLUT1 and Glycolytic Enzyme Complexes

### 9.1. Targeting GLUT1: Pharmacological Strategies

Several data sets exist regarding the pharmacological targeting of the converging regulatory nodes that govern GLUT1 subcellular localisation at PM in BC cells. Although no therapies have been approved so far, of particular interest are those agents in clinical development that target oncogenic pathways able to promote or stabilize GLUT1 at PM in BC (Table 4) and preclinical selective- and pan-GLUT1-targeting agents (Table 5). Identifying tissue-specific central nodes governing GLUT1 membrane exposure and trafficking is of considerable importance, as this could broaden the therapeutic window of systemic GLUT1 inhibition.

#### 9.1.1. Targeting GLUT1 Plasma-Membrane Stabilization

The PI3K/AKT axis is among the most clinically targeted pathway in BC to have a documented effect on GLUT1 membrane residency [126]. Alpelisib (Piqray^TM^), a PI3Kα-selective inhibitor approved for PIK3CA-mutated HR+/HER2− metastatic BC [127], and capivasertib (Truqap^TM^), a pan-AKT inhibitor approved in the same setting [128], represent the most clinically advanced agents operating on this axis; however, their effects on GLUT1 subcellular localization in BC have not been assessed in clinical studies. In metastatic BC harbouring PTEN loss, combined PIK3CA/AKT1/PTEN alterations occur in approximately 25–30% of cases [129]. In this setting, AKT inhibitors are preferred over isoform-specific PI3K inhibitors, as PTEN-null tumours show resistance to PIK3CA-targeted agent BYL719 [130]. Ipatasertib, another pan-AKT inhibitor, added to first-line paclitaxel showed an improvement in Progression-Free Survival (PFS) in PIK3CA/AKT1/PTEN-altered TNBC in the phase II LOTUS trial [131]. Unfortunately, the phase III IPATunity130 trial in biomarker-selected TNBC did not confirm the benefit of the Ipatasertib-plus-Paclitaxel regimen, underscoring the complexity of PI3K/AKT pathway targeting in this BC subtype [132]. An additional clinically relevant node is the mutp53/RhoA/ROCK axis (Table 4): tumour-associated mutant p53 promotes GLUT1 translocation to the PM through RhoA/ROCK activation, driving the Warburg effect and enhancing the glycolytic flux in cancer cells [107]. ROCK inhibitors such as fasudil have demonstrated preclinical activity against BC cell migration, though none has yet received FDA approval for oncological indications [133]. APR-246 (eprenetapopt) restores wild-type p53 conformation in mutp53-expressing tumour cells and suppresses mutp53 gain-of-function activities [134]. It has entered clinical trials in solid tumours [135]. APR-246 represents a mechanistically plausible, but as yet experimentally unverified, indirect strategy to reduce mutp53-driven GLUT1 PM accumulation.

**Table 4:** Agents in clinical development targeting oncogenic pathways able to promote or stabilize GLUT1 at plasma membrane in BC.

Target	Compound	BC-Subtype	Evidences	Ref.
PI3K/AKT	Alpelisib (Piqray^TM^)	HR+/HER2− metastatic	PI3Kα-selective inhibitor	[127]
	Capivasertib (Truqap^TM^)		pan-AKT inhibitor	[128]
	Ipatasertib + Paclitaxel	PTEN-altered TNBC	pan-AKT inhibitor	[131]
TXNIP	Fulvestrant	HR+, HER2−	SERD	[136]
	Fulvestrant + Palbociclib		combined	[137]
	Fulvestrant + Capivasertib	HR+	combined	[128]
	Inavolisib plus Fulvestrant + Palbociclib	*PIK3CA*-mutated, HR+, HER2−	combined	[138]
RhoA/ROCK	Fasudil	TNBC	ROCK inhibitor	[133]
p53	APR-246 (eprenetapopt)	mutp53	suppresses mutp53 GOF activities	[134]

#### 9.1.2. Targeting GLUT1 Trafficking

Of note is the role of TXNIP, given its involvement in the regulatory node that governs GLUT1 endocytosis and degradation (see Section 2 and Section 7.1). TXNIP is epigenetically silenced in aggressive BC subtypes [100,139]. Its repression is detrimental in BC cells and has a prognostic and predictive power in human BCs [100,101]. TXNIP expression can be indirectly restored by Fulvestrant (Table 4), a selective ER degrader (SERD), through relief of estrogen-driven transcriptional suppression, with TXNIP knockdown shown to impair fulvestrant efficacy in BC models [100]. Fulvestrant has received multiple FDA approvals for HR-positive, HER2-negative BC, initially as monotherapy (2002; NORTH AMERICAN and EUROPEAN phase III trials) [136] and subsequently in combination with targeted agents for endocrine-resistant settings [128,137,138] (Table 4). Taken together, these findings suggest TXNIP as a candidate stratification biomarker—prognostic in aggressive BC subtypes and potentially predictive of fulvestrant response—warranting prospective clinical validation before translational application.

#### 9.1.3. Targeting GLUT1 Activity

Among the agents in preclinical development (Table 5), BAY-876 is the most selective GLUT1 inhibitor identified. It has demonstrated growth inhibitory effects specifically in RB1-positive TNBC cell lines and patient-derived samples. Moreover, the efficacy of pharmacological inhibition of GLUT1 with BAY-876 is limited to a subset of TNBC cells displaying high glycolytic and lower oxidative phosphorylation (OXPHOS) rates [140]. This could indicate RB1 as a molecular biomarker that might identify patients most likely to benefit from therapies targeting this axis. Beyond BAY-876, several other GLUT1-targeting agents have demonstrated preclinical activity specifically in BC models. Among synthetic inhibitors, WZB117 resensitizes doxorubicin-resistant Luminal A BC cells to doxorubicin [141], potentiates radiation efficacy [142], and exerts synergistic cytotoxicity with AKT inhibitors by inhibiting GLUT1 in BC cells [143]. Natural polyphenols with documented GLUT1 inhibitory activity in BC cells include resveratrol, which inhibits GLUT1-mediated glucose uptake [144], and kaempferol, which inhibits GLUT1-dependent 3H-deoxy-D-glucose uptake in BC cells [145].

Among pan-GLUT inhibitors (Table 5), Glutor lacks BC-specific experimental evidence and has been characterized primarily in thymoma models [146]. Collectively, these studies highlight GLUT1 as a valuable metabolic vulnerability in BC, although none has entered clinical development. The absence of GLUT1-targeting agents in BC emphasises the opportunity for novel degradation strategies or alternatives that redirect membrane proteins towards lysosomal degradation, such as LYsosome-TArgeting Chimeras (LYTACs) [147].

Given the systemic dependence on GLUT1, its inhibition at the systemic level remains difficult to restrict to tumour tissue using conventional approaches.

**Table 5:** Agents in pre-clinical development targeting GLUT1 in BC.

Target	Compound	BC-Subtype	Evidence	Ref.
GLUT1	BAY-876	RB1 + TNBC	GLUT1 inhibitor	[140]
	WZB117	Luminal A	Overcomes doxorubicin resistance	[141]
		Luminal A, TNBC	Overcomes radio resistance	[142]
	WZB117+ MK-2206	Luminal A, TNBC	Synergistic Cytotoxic Effect	[143]
	Resveratrol	Luminal A, TNBC	inhibits GLUT1-mediated glucose uptake	[144]
	kaempferol	Luminal A	inhibits GLUT1-dependent 3H-deoxy-D-glucose	[145]

### 9.2. Targeting Glycolytic Enzymes: Pharmacological Strategies

A strategic approach for BC treatment may be to target glucosome-forming enzymes, either to inhibit their activity or to disrupt glucosome structure. Although no FDA-approved therapies targeting HK2, PFKL, and PKM2 are currently available, several candidates are currently in the preclinical phase. Given the properties of the glucosome, targeting it with drugs that affect the biochemical features of MLOs may be another promising approach to dissolve these membrane-less structures. While no therapies have yet been approved, we focus on strategies to block MLO formation or to reverse the mechanisms underlying their assembly. Some examples are well reviewed in [148].

#### 9.2.1. Targeting Enzymatic Activity

HK2 plays an important role in the first step of glycolysis because it catalyses the reaction that produces glucose-6-phosphate (G6P) starting from glucose [92]. 3-bromopyruvic acid (3-BrPA), an analogue of pyruvic acid [149,150], and 2- Deoxy-D-Glucose (2-DG), an analogue of glucose [151], act as glycolysis and HK2 inhibitors. 3-BrPA inhibits cell proliferation, through c-Myc and HK2 downregulation, leading to TXNIP upregulation (see Section 7.1), thus inhibiting glycolysis and promoting mitochondria-mediated apoptosis in TNBC cell lines [150]. Moreover, it has been reported to induce senescence in both luminal A and TNBC cell lines [149]. Additionally, in combination with 2-DG and photodynamic therapy, it can inhibit migration and proliferation of TNBC cells [152]. 2-DG has been reported to inhibit glycolysis and has been tested both in animal models and in clinical trials [151]. However, 2-DG regimens may lead to resistance [153]. Nevertheless, when used together with Compound C, an AMPK inhibitor, the effect of 2-DG on ATP production is reinforced [151]. In addition, in a recent study, a PROteolysis-TArgeting Chimera (PROTAC) system to target HK2 has also been proposed [154]. Honokiol, a natural biphenolic compound, counteracts HK2-dependent glycolysis and tumour growth by promoting HIF-1α ubiquitination and degradation, thereby suppressing HK2 expression and aerobic glycolysis in BC [155].

Research into PFK targeting has mainly focused on PFKFB3, the key enzymatic regulator of PFK, aiming to block the tumour glucose supply, but only at the pre-clinical level. The only compound to have reached human clinical testing, PFK-158, stalled at Phase I with no published efficacy data or regulatory progress (NCT02044861). Studies in cell lines have identified several molecules with inhibitory effects on PFK activity. For example, acetylsalicylic acid (aspirin) has been reported to inhibit PFK activity in luminal A BC cells, promoting its conversion from an active tetramer to inactive dimers [156].

Regarding PKM2 inhibitors, Shikonin and Alkannin, a class of necroptosis inducers, have been investigated as selective inhibitors [157,158]. These two natural compounds are reported to inhibit glycolysis [158] and to sensitise TNBC cells to paclitaxel, an effect associated with inhibition of ERK, AKT and p70S6 kinase [157].

#### 9.2.2. Targeting Glucosome Assembly and Metabolic Plasticity Across Breast Cancer Subtypes

Clotrimazole (see Section 8.1), used either alone or in combination with imatinib (a tyrosine kinase inhibitor (TKI)), is reported to inhibit proliferation, ATP production and PFK expression in T47D BC cells, as well as PFK enzymatic activity [159,160]. Whether Clotrimazole directly inhibits the formation of GE condensates is still unclear. Alternative therapeutic strategies targeting PFKL in other cancer types are well reviewed in [161].

Importantly, inhibiting glycolysis does not simply disrupt tumour metabolism but can also induce a compensatory shift toward OXPHOS. Accordingly, breast cancer cell heterogeneity further complicates the consequences of such targeting. The literature reports that basal subtypes/TNBC favour glycolysis, while luminal subtypes rely more on mitochondrial pathways. Basal-like BC cell lines/TNBC cells exhibit increased glycolytic activity and impaired mitochondrial respiration due to alterations in key electron transport chain (ETC) proteins and increased oxidative stress, whereas Luminal subtypes are more dependent on OXPHOS for ATP production.

These findings suggest that the metabolic behaviour of BC cells may differ across BC subtypes due to their heterogeneity. As such, glycolysis could be considered the preferred metabolic pathway to target in TNBC subtypes [162]. Indeed, combined treatment with 2-DG and OXPHOS inhibitors showed significant efficacy, inducing cell death in BC cells, particularly TNBC cells [163,164].

## 10. What We Know and What Remains Unknown of GLUT1/Glycolytic Assemblies

Current evidence supports the existence of spatially organised glycolytic assemblies in BC and shows that GLUT1 trafficking, GE localisation, cytoskeletal association, and glucosome formation can be dynamically regulated [11,20,109,110,111,118,119,120]. However, these observations do not yet establish a comprehensive physical interactome connecting GLUT1 to individual GEs. In particular, it remains unclear whether GLUT1 directly interacts with rate-limiting GEs such as HK2, PFK1/PFKL, or PKM2, or whether their functional coupling is mediated primarily through adaptor proteins, cytoskeletal components, membrane-associated signaling complexes, or shared condensate environments. The stoichiometry and structural interfaces of these putative assemblies are also largely unresolved.

Several additional questions remain open (Table 6). First, the extent to which GLUT1 and GE interactions differ among BC subtypes has not been systematically investigated. Second, the temporal dynamics of these interactions during glucose stimulation, GF signaling, hypoxia, migration, and cell-cycle progression remain poorly characterized. Third, it is unknown whether the molecular assemblies observed in cultured BC cells are quantitatively and structurally preserved in primary human tumours. Finally, although glucosomes display several properties compatible with dynamic biomolecular condensates, it remains unclear whether all glucosome populations arise through bona fide LLPS or instead represent other forms of multienzyme clustering.

These unresolved questions define a major experimental opportunity that can be addressed by integrating in situ XL-MS, proximity labelling, quantitative imaging, structural proteomics, and functional metabolic assays. Such approaches could address the unresolved physical architecture of GLUT1 and GE assemblies and determine where, when, and under which cellular conditions these interactions occur. Table 6 summarises these conceptual points about the GLUT1 and GE interactome in BC.

## 11. Conclusions

With this review, we build upon the traditional model—in which GLUT1 and GE expression levels are considered primary drivers of glycolytic flux and the tumour phenotype—by highlighting emerging evidence for a complementary, expression-independent regulatory layer based on their spatial organization in BC. In addition, some reviews in this field explicitly link clinical significance to the biophysics of biomolecular condensate formation. The discovery of MLOs has expanded our understanding of intracellular compartmentalization, revealing that the spatial organization of cellular processes does not rely solely on lipid bilayers [64]. Among MLOs, the glucosome is one example [11] (Section 4.4). Most of the mechanisms discussed here—including glucosome assembly and GLUT1 PM stabilization—are not unique to BC [74,109]. In the context of BC, the ability to dynamically remodel these metabolic assemblies, centred around glucose entry via transporters such as GLUT1, reveals an additional, nuanced dimension of metabolic control. This spatial regulation of GEs is observed across multiple malignancies (Table 3): PFKL-driven bio-condensates have been reported in hepatocellular carcinoma under hypoxic stress [74], and GLUT1 PM stabilization has been described in glioblastoma [109]. This cross-tumour validation supports a broader principle of metabolic regulation rather than a BC-specific trait. Conversely, based on current evidence (Table 3), the association between phSer226-GLUT1 localization and BC recurrence remains a BC-specific biomarker, with no cross-tumour validation identified to date in the literature [112]. These insights should be evaluated alongside the inherent limitations of our review. The underlying evidence base remains heterogeneous: while certain clinical observations are supported by large patient cohorts (Section 2 and Section 3), other mechanisms rely primarily on in vitro imaging in a limited number of cell lines and fluorescently tagged overexpression systems (Section 7 and Section 8). Furthermore, the assembly of a glycolytic condensate does not directly translate to enhanced metabolic flux, given its complex relationship with cell cycle status and invasive potential; functional validation in primary tumour tissues remains largely unaddressed (Table 6). From a methodological perspective, this review highlights the utility of XL-MS as a valuable tool to capture transient PPIs within both membrane and membrane-less compartments, helping to bridge a critical experimental gap. Deciphering the precise PPIs that govern the assembly and disassembly of these glycolytic metabolons will not only refine our understanding of the Warburg effect, but may also unveil novel therapeutic vulnerabilities to counteract metabolic reprogramming and treatment resistance in oncology. Nonetheless, no GLUT1- or GE-targeted agent specifically designed against the spatial mechanisms described here has yet reached clinical testing. Finally, the TXNIP/estrogen/fulvestrant axis rests on a stratified body of evidence. While TXNIP repression is a well-established pan-cancer phenomenon [165]—documented across lymphoma [166], colorectal [167], lung [168], and hepatic tumors [169]—the specific estrogen-driven regulatory circuit linking TXNIP to GLUT1 trafficking, as well as its impact on fulvestrant efficacy, has so far been characterized exclusively in BC [100]. In this review, we explore whether TXNIP, beyond its general tumour-suppressive action [165], exerts a tissue-specific regulatory function that underpins its clinical relevance, given that fulvestrant is an FDA-approved agent already used in HR-positive BC [136]. Ultimately, as an increasing number of metabolic pathways are shown to utilize MLO-mediated spatial control, biomolecular condensation should no longer be viewed as an isolated phenomenon, but rather as a fundamental potential axis to routinely account for when studying metabolic regulation in health and disease.

## Figures and Tables

**Figure 1:**
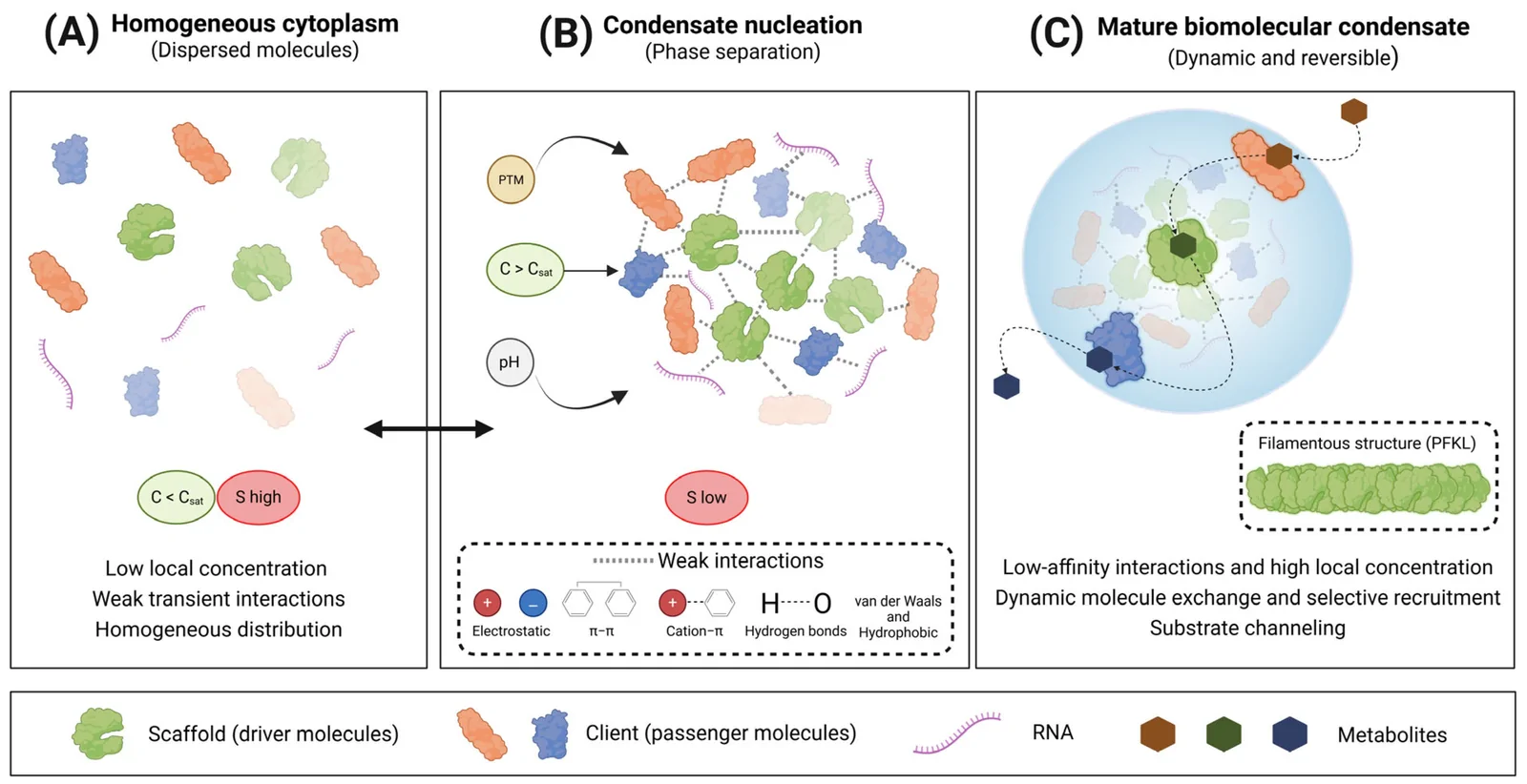
Formation and maturation of biomolecular condensates through liquid–liquid phase separation. (**A**) In the homogeneous cytoplasm, scaffold (green), client (orange/blue), and RNA (magenta) molecules are dispersed at low local concentration (C) (below the saturation concentration (Csat)) and interact with each other through transient interactions. The entropy (S) of the system is high. (**B**) Condensate nucleation can be triggered by PTMs, an increase in concentration (C), or by pH change. The molecules engage in multivalent weak interactions (grey dotted line), i.e., electrostatic, π-π, cation-π, hydrogen bonding, van der Waals and hydrophobic interactions. The entropy (S) is lower. The change in system conditions can trigger A-to-B or B-to-A transition (double arrow). (**C**) Scaffold molecules can assemble into defined 3D structures (i.e., PFKL into filamentous structures). The mature biomolecular condensate exhibits dynamic and reversible exchange of components with the surrounding environment, in particular substrate channelling (metabolites, coloured hexagons) among interior components.

**Figure 2:**
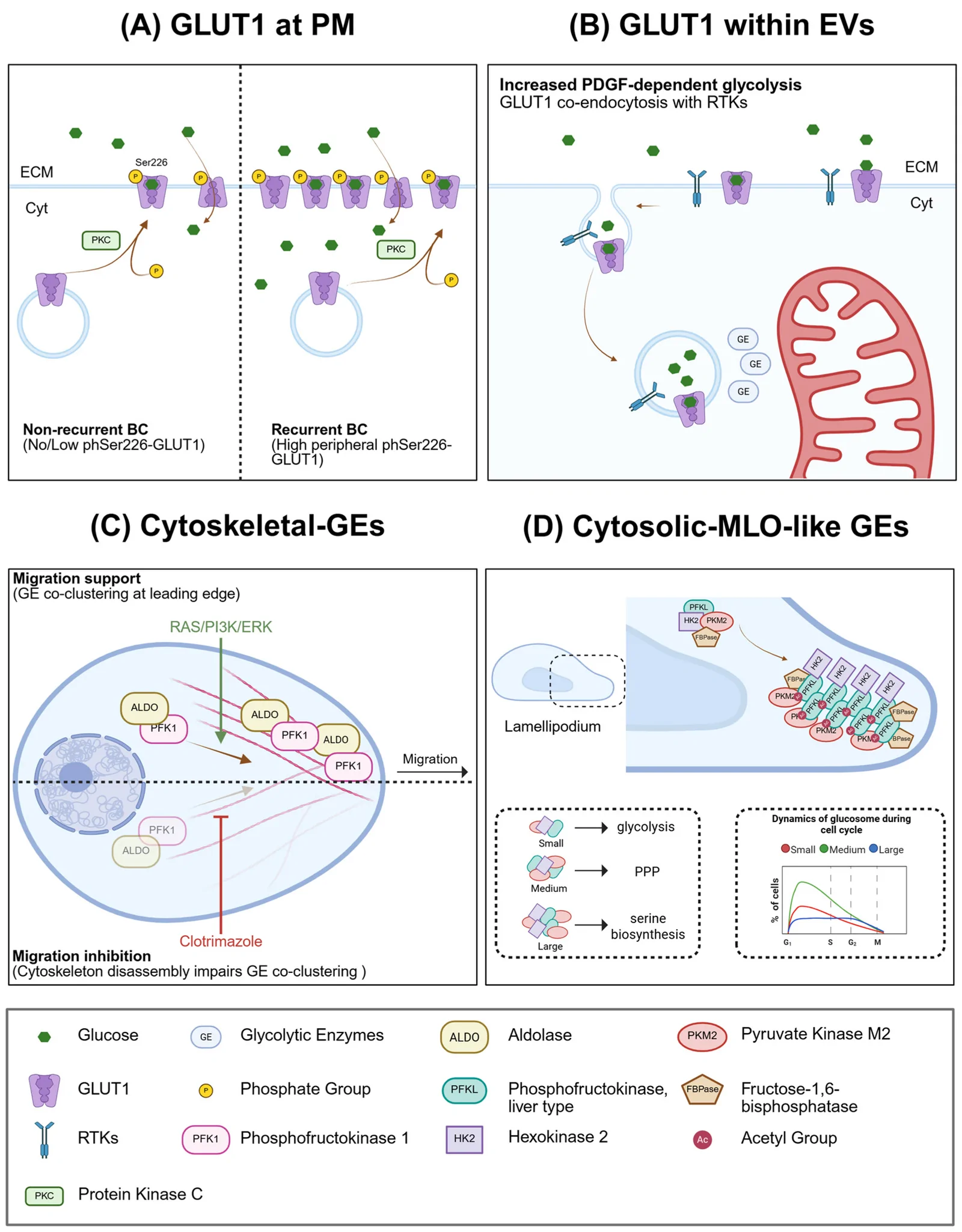
GLUT1 and GE subcellular localization. (**A**) Protein Kinase C (PKC) phosphorylates GLUT1 on Ser226 to enhance its cell surface localization and glucose uptake. phSer226-GLUT1 predicts BC recurrence. (**B**) Glucose-loaded endocytic vesicle with GLUT1 and RTKs bringing glucose near GEs, localized adjacent to mitochondria. (**C**) The relocalization of GEs from the cytosol to the PM (Ras/PI3K/ERK-dependent) increases BC cell migration. Clotrimazole treatment stimulates GE detachment from actin fibers, inhibiting their association with the PM. (**D**) PFKL forms variable-sized cytoplasmic clusters with GEs to adapt invasive behaviour and metabolic needs, and to coordinate the cell-cycle.

**Table 1:** Comparative overview of methods for PPI analysis.

Method	Throughput	PPI Type	Spatial Information	Native Context	Key Strengths	Main Limitations
Y2H	High	Binary, stable	No	No	Scalable; maps large interactomes; historical cornerstone	Not suitable for complexes, membrane proteins, and compartmentalized PPIs
Co-IP	Low– Medium	Stable	No	Yes	Biochemical evidence; robust for stable complexes	Misses transient PPIs; detergent sensitivity; requires antibodies
AP-MS	Medium	Stable, weak (some)	No	Partial	Defines complex composition; proteome-wide applicability	Loss of labile interactions; lysis disrupts assemblies
FRET/BRET	Low– Medium	Transient, weak, proximity-based	High (nano-meter proximity)	Yes	Real-time monitoring in living cells; excellent for dynamics	Requires tagging; limited to pairwise interactions
PLA	Medium	Weak, transient	High (sub-cellular resolution)	Yes	Detects endogenous PPIs in intact cells/tissues	Semi-quantitative; dependent on antibody specificity
Proximity labeling (BioID, TurboID, APEX2)	Medium–High	Weak, transient, compartment-specific, proximity-based	Indirect (labelling radius)	Yes	Captures transient PPIs; excellent for membrane proteins and condensates	Requires engineered constructs; proximity ≠ direct interaction
SPR	Low	Weak, transient (Kinetics)	No	No	Quantitative binding kinetics; high sensitivity	In vitro; requires purified proteins
XL-MS	Medium	Stable, weak, transient	High (distance constraints)	Yes	Resolves topology; preserves transient and compartmentalized PPIs; ideal for metabolons and MLOs	Coverage uneven; requires optimization; partial structural view
LiP-MS	Medium	Conformational/structural	Indirect	Yes	Detects proteome-wide structural rearrangements; ideal for dynamic/disordered proteins	Does not directly identify binding partners
FRAP/FLIM/FLIM-FRET	Low	Assembly dynamics (not direct PPIs)	High (spatio-temporal dynamics)	Yes	Measures assembly turnover, mobility, and reconfiguration	No direct evidence of physical PPIs
Cryo-EM	Low	Stable	Very high (near-native)	Yes	Resolves endogenous assemblies; captures heterogeneity	Requires abundant material; not suited for highly dynamic complex
AI-driven structural prediction (AlphaFold3)	High	Stable, weak (interface prediction)	High (model-based)	Yes (integrated with experimental data)	Predicts interfaces and architectures of intractable assemblies	Predictive; requires experimental validation

**Table 2:** Oncogenic pathways able to promote or stabilize GLUT1 at plasma membrane in BC.

Pathway	Role	BC-Subtype	Evidences	Ref.
PI3KC1-AKT	oncogenic role	Luminal A	abnormal activation of the PI3KC1-AKT pathway leads to preferential GLUT1 at PM	[103]
USP6NL/AKT	oncogenic overexpression	TNBC	High level of USP6NL causes chronic AKT activation stabilizing GLUT1 at the PM	[102]
TXNIP	oncogenic repression	TNBC	c-Myc reduces TXNIP gene expression, increasing glucose uptake	[104]
		TNBC; luminal A	miR-373 suppresses TXNIP by binding to its 3′UTR	[105]
		Luminal A	estrogen represses TXNIP expression	[106]
mutp53/RhoA/ ROCK	oncogenic mutation	TNBC; luminal A, HER2+	Mutp53 stimulates the Warburg effect through promoting GLUT1 to the PM	[107]
PTEN	oncogenic loss	Luminal A	PTEN loss impedes interaction with AKT, allowing its phosphorylation and thus promoting GLUT1 to the PM	[103]
		TNBC	PTEN loss impedes binding with SNX27 to prevent GLUT1 accumulation at the PM	[108]

**Table 3:** Evidence supporting spatial regulation of GLUT1 and GE organization in BC.

Mechanism	Molecular Process	Experimental Model	BC Subtype	Methodology	Evidence Level
GLUT1 PM stabilization	AKT-dependent GLUT1 membrane retention	BC cell lines	TNBC/ Luminal	Immunofluorescence, biochemical fractionation	Experimental
GLUT1 trafficking	TXNIP-dependent endocytosis/degradation	BC models + patient samples	TNBC/ Luminal	IF, molecular biology, clinical correlation	Experimental + clinical
Ser226GLUT1 phosphorylation	PKC-dependent PM recruitment	BC cells/ tissues	Multiple BC	Phosphoproteomics/IF/functional assays	Experimental + clinical
GE recruitment to actin	PFK1/Aldolase association with cytoskeleton	BC cells	TNBC/ Luminal	Biochemical fractionation, imaging, metabolic assays	Experimental
Glycolytic waves	F-actin-associated GE waves	BC cells	TNBC	Live-cell imaging	Experimental
Glucosome formation	PFKL-dependent multienzyme assemblies	BC cells	TNBC	FRET, FRAP, microscopy	Experimental
Glucosome size/metabolic fate	Size-dependent metabolic routing	cancer cells/ BC models	BC/ other cancer	Microscopy/metabolomics	Experimental
PFKL filamentation	Filament-dependent assembly	HCC	Other cancer	Cryo-EM/structural analysis	Experimental
GLUT1 stabilization	Membrane retention	glioblastoma	Other cancer	Imaging/biochemical assays	Experimental
LLPS principles	Phase separation, multivalency, IDRs	reconstituted/ general systems	General	Biophysical assays	Mechanistic/general

**Table 6:** What is known and what remains unknown about the GLUT1 and GE interactome in BC.

Question	What We Know	What Remains Unknown
GLUT1–GE interaction	GLUT1/GEs occupy spatially restricted, functionally coupled compartments; GEs associate with GLUT1^+^ EVs surfaces, coupling glucose transport to glycolysis [114,115,116].	Whether GLUT1 directly binds GEs in BC cells, or whether coupling is indirect.
GE–GE Interactions and glucosome assembly	Several GEs can physically associate with each other. PFKL functions as a central component of glucosome assemblies and interacts with FBPase; PFKL also co-clusters with HK2 and PKM2 in TNBC cells [11,73,74,123].	Whether these GE–GE interactions are organized into a defined complex, and whether their composition and stoichiometry are conserved across BC subtypes.
Structural Interfaces and stoichiometry	Select GE–GE interactions have experimental support (PFKL–FBPase); multienzyme glycolytic assemblies vary in size/composition with metabolic and cell-cycle state [11,73,122,124,125].	Structural interfaces linking GLUT1 to GEs, and the molecular stoichiometry of multienzyme assemblies.
Temporal dynamics	GLUT1 trafficking is dynamically regulated by oncogenic signaling and endocytic processes; glucosomes show dynamic molecular exchange and cell-cycle-dependent assembly; F-actin glycolytic waves are dynamically regulated during migration [11,100,102,118,120,121,124,125].	Temporal dynamics of GLUT1–GE interactions, including lifetime, assembly/disassembly kinetics, and response to glucose, growth factors, hypoxia, or metabolic stress.
Subtype specificity	Spatial regulation of GLUT1/GEs documented across contexts: PFKL-containing clusters in TNBC; GLUT1 PM localization and regulatory pathways investigated in TNBC and luminal models [102,103,108,112,122,123].	Whether GLUT1/GE interaction networks differ across BC subtypes and how their architecture contributes to phenotype.
Clinical relevance	Peripheral phSer226-GLUT1 localization associated with aggressive BC and clinical outcome; GLUT1/GE localization and activity linked to BC aggressiveness and migration [112,117,119].	Whether GLUT1/GE architecture in experimental models or primary tumours has prognostic/predictive value.

## Data Availability

No new data were created or analyzed in this study. Data sharing is not applicable to this article.

## References

[1] Vander Heiden M.G., Cantley L.C., Thompson C.B. (2009). Understanding the Warburg effect: The metabolic requirements of cell proliferation. Science.

[2] Hanahan D., Weinberg R.A. (2011). Hallmarks of cancer: The next generation. Cell.

[3] Mitaishvili E., Feinsod H., David Z., Shpigel J., Fernandez C., Sauane M., de la Parra C. (2024). The Molecular Mechanisms behind Advanced Breast Cancer Metabolism: Warburg Effect, OXPHOS, and Calcium. Front. Biosci..

[4] Harbeck N., Penault-Llorca F., Cortes J., Gnant M., Houssami N., Poortmans P., Ruddy K., Tsang J., Cardoso F. (2019). Breast cancer. Nat. Rev. Dis. Primers.

[5] Sørlie T., Perou C.M., Tibshirani R., Aas T., Geisler S., Johnsen H., Hastie T., Eisen M.B., Van De Rijn M., Jeffrey S.S. (2001). Gene expression patterns of breast carcinomas distinguish tumor subclasses with clinical implications. Proc. Natl. Acad. Sci. USA.

[6] Reis-Filho J.S., Tutt A.N.J. (2008). Triple negative tumours: A critical review. Histopathology.

[7] de Mooij C.M., Ploumen R.A.W., Nelemans P.J., Mottaghy F.M., Smidt M.L., van Nijnatten T.J.A. (2023). The influence of receptor expression and clinical subtypes on baseline [18F]FDG uptake in breast cancer: Systematic review and meta-analysis. EJNMMI Res..

[8] Wang Z., Jiang Q., Dong C. (2020). Metabolic reprogramming in triple-negative breast cancer. Cancer Biol. Med..

[9] Mueckler M., Thorens B. (2013). The SLC2 (GLUT) family of membrane transporters. Mol. Asp. Med..

[10] Lunt S.Y., Vander Heiden M.G. (2011). Aerobic glycolysis: Meeting the metabolic requirements of cell proliferation. Annu. Rev. Cell Dev. Biol..

[11] Kohnhorst C.L., Kyoung M., Jeon M., Schmitt D.L., Kennedy E.L., Ramirez J., Bracey S.M., Luu B.T., Russell S.J., An S. (2017). Identification of a multienzyme complex for glucose metabolism in living cells. J. Biol. Chem..

[12] Attanasio F., Caldieri G., Giacchetti G., van Horssen R., Wieringa B., Buccione R. (2011). Novel invadopodia components revealed by differential proteomic analysis. Eur. J. Cell Biol..

[13] Santorelli L., Costanzo M., Petrosino S., Santoro M., Caterino M., Ruoppolo M., Grumati P. (2025). Cross-Linking Mass Spectrometry to Capture Protein Network Dynamics of Cell Membranome. Tissue Proteomics: Methods and Protocols.

[14] Piersimoni L., Kastritis P.L., Arlt C., Sinz A. (2022). Cross-Linking Mass Spectrometry for Investigating Protein Conformations and Protein-Protein Interactions-A Method for All Seasons. Chem. Rev..

[15] Kang S.S., Chun Y.K., Hur M.H., Lee H.K., Kim Y.J., Hong S.R., Lee J.H., Lee S.G., Park Y.K. (2002). Clinical significance of glucose transporter 1 (GLUT1) expression in human breast carcinoma. Jpn. J. Cancer Res..

[16] Hussein Y.R., Bandyopadhyay S., Semaan A., Ahmed Q., Albashiti B., Jazaerly T., Nahleh Z., Ali-Fehmi R. (2011). Glut-1 Expression Correlates with Basal-like Breast Cancer. Transl. Oncol..

[17] Deng Y., Zou J., Deng T., Liu J. (2018). Clinicopathological and prognostic significance of GLUT1 in breast cancer: A meta-analysis. Medicine.

[18] Brown R.S., Wahl R.L. (1993). Overexpression of glut-1 glucose transporter in human breast cancer an immunohistochemical study. Cancer.

[19] Wang J., Ye C., Chen C., Xiong H., Xie B., Zhou J., Chen Y., Zheng S., Wang L. (2017). Glucose transporter GLUT1 expression and clinical outcome in solid tumors: A systematic review and meta-analysis. Oncotarget.

[20] Petrosino S., Grumati P. (2026). Autophagy and GLUT1 trafficking: An overview of molecular mechanisms. Am. J. Physiol. Cell Physiol..

[21] Orofiamma L.A., Vural D., Antonescu C.N. (2022). Control of cell metabolism by the epidermal growth factor receptor. Biochim. Biophys. Acta. Mol. Cell Res..

[22] Zhu P., Liu G., Wang X., Lu J., Zhou Y., Chen S., Gao Y., Wang C., Yu J., Sun Y. (2022). Transcription factor c-Jun modulates GLUT1 in glycolysis and breast cancer metastasis. BMC Cancer.

[23] Chen C., Pore N., Behrooz A., Ismail-Beigi F., Maity A. (2001). Regulation of glut1 mRNA by hypoxia-inducible factor-1: Interaction between H-ras and hypoxia. J. Biol. Chem..

[24] Liu L., Bai J., Hu L., Jiang D. (2023). Hypoxia-mediated activation of hypoxia-inducible factor-1α in triple-negative breast cancer: A review. Medicine.

[25] Osthus R.C., Shim H., Kim S., Li Q., Reddy R., Mukherjee M., Xu Y., Wonsey D., Lee L.A., Dang C.V. (2000). Deregulation of glucose transporter 1 and glycolytic gene expression by c-Myc. J. Biol. Chem..

[26] Stine Z.E., Walton Z.E., Altman B.J., Hsieh A.L., Dang C.V. (2015). MYC, Metabolism, and Cancer. Cancer Discov..

[27] Abu-Remaileh M., Aqeilan R.I. (2014). Tumor suppressor WWOX regulates glucose metabolism via HIF1α modulation. Cell Death Differ..

[28] Pospiech K., Płuciennik E., Bednarek A.K. (2018). WWOX Tumor Suppressor Gene in Breast Cancer, a Historical Perspective and Future Directions. Front. Oncol..

[29] Schwartzenberg-Bar-Yoseph F., Armoni M., Karnieli E. (2004). The tumor suppressor p53 down-regulates glucose transporters GLUT1 and GLUT4 gene expression. Cancer Res..

[30] Harami-Papp H., Pongor L.S., Munkácsy G., Horváth G., Nagy Á.M., Ambrus A., Hauser P., Szabó A., Tretter L., Győrffy B. (2016). TP53 mutation hits energy metabolism and increases glycolysis in breast cancer. Oncotarget.

[31] Pinheiro C., Sousa B., Albergaria A., Paredes J., Dufloth R., Vieira D., Schmitt F., Baltazar F. (2011). GLUT1 and CAIX expression profiles in breast cancer correlate with adverse prognostic factors and MCT1 overexpression. Histol. Histopathol..

[32] Wellberg E.A., Johnson S., Finlay-Schultz J., Lewis A.S., Terrell K.L., Sartorius C.A., Abel E.D., Muller W.J., Anderson S.M. (2016). The glucose transporter GLUT1 is required for ErbB2-induced mammary tumorigenesis. Breast Cancer Res. BCR.

[33] Labak C.M., Wang P.Y., Arora R., Guda M.R., Asuthkar S., Tsung A.J., Velpula K.K. (2016). Glucose transport: Meeting the metabolic demands of cancer, and applications in glioblastoma treatment. Am. J. Cancer Res..

[34] Oh S., Kim H., Nam K., Shin I. (2017). Glut1 promotes cell proliferation, migration and invasion by regulating epidermal growth factor receptor and integrin signaling in triple-negative breast cancer cells. BMB Rep..

[35] Ishfaq M., Bashir N., Riaz S.K., Manzoor S., Khan J.S., Bibi Y., Sami R., Aljahani A.H., Alharthy S.A., Shahid R. (2022). Expression of HK2, PKM2, and PFKM Is Associated with Metastasis and Late Disease Onset in Breast Cancer Patients. Genes.

[36] Palmieri D., Fitzgerald D., Shreeve S.M., Hua E., Bronder J.L., Weil R.J., Davis S., Stark A.M., Merino M.J., Kurek R. (2009). Analyses of resected human brain metastases of breast cancer reveal the association between up-regulation of hexokinase 2 and poor prognosis. Mol. Cancer Res. MCR.

[37] Yeerken D., Hong R., Wang Y., Gong Y., Liu R., Yang D., Li J., Fan J., Chen J., Zhang W. (2020). PFKP is transcriptionally repressed by BRCA1/ZBRK1 and predicts prognosis in breast cancer. PLoS ONE.

[38] Semenza G.L. (2010). HIF-1: Upstream and downstream of cancer metabolism. Curr. Opin. Genet. Dev..

[39] Zhang H.-S., Du G.-Y., Zhang Z.-G., Zhou Z., Sun H.-L., Yu X.-Y., Shi Y.-T., Xiong D.-N., Li H., Huang Y.-H. (2018). NRF2 facilitates breast cancer cell growth via HIF1ɑ-mediated metabolic reprogramming. Int. J. Biochem. Cell Biol..

[40] Umar S.M., Kashyap A., Kahol S., Mathur S.R., Gogia A., Deo S.V.S., Prasad C.P. (2020). Prognostic and therapeutic relevance of phosphofructokinase platelet-type (PFKP) in breast cancer. Exp. Cell Res..

[41] Zancan P., Sola-Penna M., Furtado C.M., Da Silva D. (2010). Differential expression of phosphofructokinase-1 isoforms correlates with the glycolytic efficiency of breast cancer cells. Mol. Genet. Metab..

[42] Moon J.-S., Kim H.E., Koh E., Park S.H., Jin W.-J., Park B.-W., Park S.W., Kim K.-S. (2011). Krüppel-like factor 4 (KLF4) activates the transcription of the gene for the platelet isoform of phosphofructokinase (PFKP) in breast cancer. J. Biol. Chem..

[43] Kim N.H., Cha Y.H., Lee J., Lee S.-H., Yang J.H., Yun J.S., Cho E.S., Zhang X., Nam M., Kim N. (2017). Snail reprograms glucose metabolism by repressing phosphofructokinase PFKP allowing cancer cell survival under metabolic stress. Nat. Commun..

[44] Han X., Ren C., Lu C., Qiao P., Yang T., Yu Z. (2022). Deubiquitination of MYC by OTUB1 contributes to HK2 mediated glycolysis and breast tumorigenesis. Cell Death Differ..

[45] Han X., Ren C., Lu C., Jiang A., Wang X., Liu L., Yu Z. (2024). Phosphorylation of USP27X by PIM2 promotes glycolysis and breast cancer progression via deubiquitylation of MYC. Oncogene.

[46] Gao Y., Yang Y., Yuan F., Huang J., Xu W., Mao B., Yuan Z., Bi W. (2017). TNFα-YAP/p65-HK2 axis mediates breast cancer cell migration. Oncogenesis.

[47] Kim N.H., Sung N.J., Youn H.-S., Park S.-A. (2020). Gremlin-1 activates Akt/STAT3 signaling, which increases the glycolysis rate in breast cancer cells. Biochem. Biophys. Res. Commun..

[48] Huang Y.-H., Tu Y.-C., Chien P.-J., Lee A.-J., Lin C.-L., Li S.-T., Lee Y.-C., Chang W.-W. (2026). Targeting SRC/STAT3 Signaling Impairs Cancer Stem Cell Activity by Downregulation of Hexokinase-2 in Radioresistant Triple-Negative Breast Cancer Cells. Oncol. Res..

[49] Liu Y.-H., Wei X.-L., Hu G.-Q., Wang T.-X. (2015). Quinolone-indolone conjugate induces apoptosis by inhibiting the EGFR-STAT3-HK2 pathway in human cancer cells. Mol. Med. Rep..

[50] Pandkar M.R., Raveendran A., Biswas K., Mutnuru S.A., Mishra J., Samaiya A., Malys T., Mitrophanov A.Y., Sharan S.K., Shukla S. (2023). PKM2 dictates the poised chromatin state of PFKFB3 promoter to enhance breast cancer progression. NAR Cancer.

[51] Blaha C.S., Ramakrishnan G., Jeon S.-M., Nogueira V., Rho H., Kang S., Bhaskar P., Terry A.R., Aissa A.F., Frolov M.V. (2022). A non-catalytic scaffolding activity of hexokinase 2 contributes to EMT and metastasis. Nat. Commun..

[52] Li S., Gong J., Kang B., Wang Z., Ma Y., Xia X., Yan H. (2026). Targeting Glycolytic Metabolism in Cancer Therapy: Current Approaches and Future Perspectives. Cells.

[53] Huang S., Czech M.P. (2007). The GLUT4 glucose transporter. Cell Metab..

[54] Egert S., Nguyen N., Schwaiger M. (1999). Myocardial glucose transporter GLUT1: Translocation induced by insulin and ischemia. J. Mol. Cell. Cardiol..

[55] Vogt B., Mushack J., Seffer E., Häring H.U. (1991). The translocation of the glucose transporter sub-types GLUT1 and GLUT4 in isolated fat cells is differently regulated by phorbol esters. Biochem. J..

[56] Campos M., Albrecht L.V. (2023). Hitting the Sweet Spot: How Glucose Metabolism Is Orchestrated in Space and Time by Phosphofructokinase-1. Cancers.

[57] Alberti S., Gladfelter A., Mittag T. (2019). Considerations and Challenges in Studying Liquid-Liquid Phase Separation and Biomolecular Condensates. Cell.

[58] Banani S.F., Lee H.O., Hyman A.A., Rosen M.K. (2017). Biomolecular condensates: Organizers of cellular biochemistry. Nat. Rev. Mol. Cell Biol..

[59] Franzmann T.M., Alberti S. (2019). Prion-like low-complexity sequences: Key regulators of protein solubility and phase behavior. J. Biol. Chem..

[60] Li P., Banjade S., Cheng H.-C., Kim S., Chen B., Guo L., Llaguno M., Hollingsworth J.V., King D.S., Banani S.F. (2012). Phase transitions in the assembly of multivalent signalling proteins. Nature.

[61] Alberti S. (2017). Phase separation in biology. Curr. Biol. CB.

[62] Banani S.F., Rice A.M., Peeples W.B., Lin Y., Jain S., Parker R., Rosen M.K. (2016). Compositional Control of Phase-Separated Cellular Bodies. Cell.

[63] Chong P.A., Forman-Kay J.D. (2016). Liquid-liquid phase separation in cellular signaling systems. Curr. Opin. Struct. Biol..

[64] Gomes E., Shorter J. (2019). The molecular language of membraneless organelles. J. Biol. Chem..

[65] Dignon G.L., Best R.B., Mittal J. (2020). Biomolecular Phase Separation: From Molecular Driving Forces to Macroscopic Properties. Annu. Rev. Phys. Chem..

[66] Roth C.M., Neal B.L., Lenhoff A.M. (1996). Van der Waals interactions involving proteins. Biophys. J..

[67] Ditlev J.A., Case L.B., Rosen M.K. (2018). Who’s In and Who’s Out-Compositional Control of Biomolecular Condensates. J. Mol. Biol..

[68] Boeynaems S., Alberti S., Fawzi N.L., Mittag T., Polymenidou M., Rousseau F., Schymkowitz J., Shorter J., Wolozin B., Van Den Bosch L. (2018). Protein Phase Separation: A New Phase in Cell Biology. Trends Cell Biol..

[69] Spolaore B., Secco L., Rocca G., Manfioletti G., Arrigoni G., Sgarra R. (2023). Proteomic tools to study phosphorylation of intrinsically disordered proteins. Expert Rev. Proteom..

[70] Secco L., Rocca G., Ingo A.M.D., Pobega E., Laera V., Manfioletti G., Arrigoni G., Sgarra R. (2026). Unveiling the unfolded proteome: Evaluation and comparison of methods for the enrichment of intrinsically disordered proteins. Protein Sci. A Publ. Protein Soc..

[71] Zhang H., Ji X., Li P., Liu C., Lou J., Wang Z., Wen W., Xiao Y., Zhang M., Zhu X. (2020). Liquid-liquid phase separation in biology: Mechanisms, physiological functions and human diseases. Sci. China Life Sci..

[72] Courchaine E.M., Lu A., Neugebauer K.M. (2016). Droplet organelles?. EMBO J..

[73] Kang H.-W., Nguyen L., An S., Kyoung M. (2024). Mechanistic insights into condensate formation of human liver-type phosphofructokinase by stochastic modeling approaches. Sci. Rep..

[74] Jin M., Fuller G.G., Han T., Yao Y., Alessi A.F., Freeberg M.A., Roach N.P., Moresco J.J., Karnovsky A., Baba M. (2017). Glycolytic Enzymes Coalesce in G Bodies under Hypoxic Stress. Cell Rep..

[75] Fuller G.G., Han T., Freeberg M.A., Moresco J.J., Ghanbari Niaki A., Roach N.P., Yates J.R., Myong S., Kim J.K. (2020). RNA promotes phase separation of glycolysis enzymes into yeast G bodies in hypoxia. ELife.

[76] Brückner A., Polge C., Lentze N., Auerbach D., Schlattner U. (2009). Yeast two-hybrid, a powerful tool for systems biology. Int. J. Mol. Sci..

[77] Bunn R.C., Jensen M.A., Reed B.C. (1999). Protein interactions with the glucose transporter binding protein GLUT1CBP that provide a link between GLUT1 and the cytoskeleton. Mol. Biol. Cell.

[78] Yang X., Cui W., Wang L., Zheng Y. (2026). A Brief Progress in Methods for Deciphering Protein-Protein Interaction Networks. Int. J. Mol. Sci..

[79] Santorelli L., Caterino M., Costanzo M. (2022). Dynamic Interactomics by Cross-Linking Mass Spectrometry: Mapping the Daily Cell Life in Postgenomic Era. OMICS A J. Integr. Biol..

[80] Gingras A.-C., Gstaiger M., Raught B., Aebersold R. (2007). Analysis of protein complexes using mass spectrometry. Nat. Rev. Mol. Cell Biol..

[81] Dunham W.H., Mullin M., Gingras A.-C. (2012). Affinity-purification coupled to mass spectrometry: Basic principles and strategies. Proteomics.

[82] Niles A.L., Dibble M.R.C., Machleidt T., Martino K., Swiatnicki M.R., Vu E.H., Schwinn M.K. (2025). Bioluminescence-based assays for quantifying endogenous protein interactions in live cells. J. Biol. Chem..

[83] Zamiati D., Abdesselem M., Nüsse O., Erard M. (2026). Quantitative FRET FLIM imaging reveals multiple interactions between proteins of the ESYT, ORP, and VAP families at the endoplasmic reticulum membrane. J. Biol. Chem..

[84] Morgan D.C., Dong Z., Kim T., Raftopoulos P., Bloomer B.J., MacMillan D.W.C. (2026). Advances in proximity labeling strategies for interactome mapping and functional interrogation. Curr. Opin. Chem. Biol..

[85] Douzi B. (2024). Surface Plasmon Resonance: A Sensitive Tool to Study Protein-Protein Interactions. Bacterial Secretion Systems: Methods and Protocols.

[86] Wu F., Minteer S. (2015). Krebs cycle metabolon: Structural evidence of substrate channeling revealed by cross-linking and mass spectrometry. Angew. Chem. Int. Ed. Engl..

[87] Boczek E.E., Fürsch J., Niedermeier M.L., Jawerth L., Jahnel M., Ruer-Gruß M., Kammer K.-M., Heid P., Mediani L., Wang J. (2021). HspB8 prevents aberrant phase transitions of FUS by chaperoning its folded RNA-binding domain. ELife.

[88] Malinovska L., Cappelletti V., Kohler D., Piazza I., Tsai T.-H., Pepelnjak M., Stalder P., Dörig C., Sesterhenn F., Elsässer F. (2023). Proteome-wide structural changes measured with limited proteolysis-mass spectrometry: An advanced protocol for high-throughput applications. Nat. Protoc..

[89] Van Leene C., Van Moortel L., De Bosscher K., Gevaert K. (2025). Exploring protein conformations with limited proteolysis coupled to mass spectrometry. Trends Biochem. Sci..

[90] Bonilla P.A., Shrestha R. (2024). FLIM-FRET Protein-Protein Interaction Assay.

[91] An S., Parajuli P., Kennedy E.L., Kyoung M. (2022). Multi-dimensional Fluorescence Live-Cell Imaging for Glucosome Dynamics in Living Human Cells. Multienzymatic Assemblies: Methods and Protocols.

[92] Lynch E.M., Hansen H., Salay L., Cooper M., Timr S., Kollman J.M., Webb B.A. (2024). Structural basis for allosteric regulation of human phosphofructokinase-1. Nat. Commun..

[93] Skalidis I., Kyrilis F.L., Tüting C., Hamdi F., Chojnowski G., Kastritis P.L. (2022). Cryo-EM and artificial intelligence visualize endogenous protein community members. Structure.

[94] Träger T.K., Tüting C., Kastritis P.L. (2024). The human touch: Utilizing AlphaFold 3 to analyze structures of endogenous metabolons. Structure.

[95] Amaral B.C., Michael A.R.M., Brodie N.I., Crowder D.A., Eiriksson K.H., Schriemer D.C. (2025). Click-linking: A cell-compatible protein crosslinking method based on click chemistry. Nat. Commun..

[96] Jiao F., Yu C., Wheat A., Chen L., Lih T.-S.M., Zhang H., Huang L. (2024). DSBSO-Based XL-MS Analysis of Breast Cancer PDX Tissues to Delineate Protein Interaction Network in Clinical Samples. J. Proteome Res..

[97] Johnson F.D., Hughes C.S., Liu A., Lockwood W.W., Morin G.B. (2023). Tandem mass tag-based thermal proteome profiling for the discovery of drug-protein interactions in cancer cells. STAR Protoc..

[98] Avril N., Menzel M., Dose J., Schelling M., Weber W., Jänicke F., Nathrath W., Schwaiger M. (2001). Glucose metabolism of breast cancer assessed by 18F-FDG PET: Histologic and immunohistochemical tissue analysis. J. Nucl. Med. Off. Publ. Soc. Nucl. Med..

[99] Aloj L., Caracó C., Jagoda E., Eckelman W.C., Neumann R.D. (1999). Glut-1 and hexokinase expression: Relationship with 2-fluoro-2-deoxy-D-glucose uptake in A431 and T47D cells in culture. Cancer Res..

[100] Park J.W., Lee S.H., Woo G.-H., Kwon H.-J., Kim D.-Y. (2018). Downregulation of TXNIP leads to high proliferative activity and estrogen-dependent cell growth in breast cancer. Biochem. Biophys. Res. Commun..

[101] Qualls-Histed S.J., Nielsen C.P., MacGurn J.A. (2023). Lysosomal trafficking of the glucose transporter GLUT1 requires sequential regulation by TXNIP and ubiquitin. IScience.

[102] Avanzato D., Pupo E., Ducano N., Isella C., Bertalot G., Luise C., Pece S., Bruna A., Rueda O.M., Caldas C. (2018). High USP6NL Levels in Breast Cancer Sustain Chronic AKT Phosphorylation and GLUT1 Stability Fueling Aerobic Glycolysis. Cancer Res..

[103] Phadngam S., Castiglioni A., Ferraresi A., Morani F., Follo C., Isidoro C. (2016). PTEN dephosphorylates AKT to prevent the expression of GLUT1 on plasmamembrane and to limit glucose consumption in cancer cells. Oncotarget.

[104] Shen L., O’Shea J.M., Kaadige M.R., Cunha S., Wilde B.R., Cohen A.L., Welm A.L., Ayer D.E. (2015). Metabolic reprogramming in triple-negative breast cancer through Myc suppression of TXNIP. Proc. Natl. Acad. Sci. USA.

[105] Chen D., Dang B.-L., Huang J., Chen M., Wu D., Xu M.-L., Li R., Yan G.-R. (2015). MiR-373 drives the epithelial-to-mesenchymal transition and metastasis via the miR-373-TXNIP-HIF1α-TWIST signaling axis in breast cancer. Oncotarget.

[106] Wang Y., Chen S. (2022). TXNIP Links Anticipatory Unfolded Protein Response to Estrogen Reprogramming Glucose Metabolism in Breast Cancer Cells. Endocrinology.

[107] Zhang C., Liu J., Liang Y., Wu R., Zhao Y., Hong X., Lin M., Yu H., Liu L., Levine A.J. (2013). Tumour-associated mutant p53 drives the Warburg effect. Nat. Commun..

[108] Shinde S.R., Maddika S. (2017). PTEN Regulates Glucose Transporter Recycling by Impairing SNX27 Retromer Assembly. Cell Rep..

[109] Zhang Z., Li X., Yang F., Chen C., Liu P., Ren Y., Sun P., Wang Z., You Y., Zeng Y.-X. (2021). DHHC9-mediated GLUT1 S-palmitoylation promotes glioblastoma glycolysis and tumorigenesis. Nat. Commun..

[110] Andrisse S., Patel G.D., Chen J.E., Webber A.M., Spears L.D., Koehler R.M., Robinson-Hill R.M., Ching J.K., Jeong I., Fisher J.S. (2013). ATM and GLUT1-S490 phosphorylation regulate GLUT1 mediated transport in skeletal muscle. PLoS ONE.

[111] Lee E.E., Ma J., Sacharidou A., Mi W., Salato V.K., Nguyen N., Jiang Y., Pascual J.M., North P.E., Shaul P.W. (2015). A Protein Kinase C Phosphorylation Motif in GLUT1 Affects Glucose Transport and is Mutated in GLUT1 Deficiency Syndrome. Mol. Cell.

[112] Kraft A.M., Petty H.R. (2020). Spatial locations of certain enzymes and transporters within preinvasive ductal epithelial cells predict human breast cancer recurrences. Am. J. Physiol. Cell Physiol..

[113] Godoy A., Ulloa V., Rodríguez F., Reinicke K., Yañez A.J., García M.d.l.A., Medina R.A., Carrasco M., Barberis S., Castro T. (2006). Differential subcellular distribution of glucose transporters GLUT1-6 and GLUT9 in human cancer: Ultrastructural localization of GLUT1 and GLUT5 in breast tumor tissues. J. Cell. Physiol..

[114] Tsutsumi R., Ueberheide B., Liang F.-X., Neel B.G., Sakai R., Saito Y. (2024). Endocytic vesicles act as vehicles for glucose uptake in response to growth factor stimulation. Nat. Commun..

[115] Wang H., Vant J.W., Zhang A., Sanchez R.G., Wu Y., Micou M.L., Luczak V., Whiddon Z., Carlson N.M., Yu S.B. (2024). Organization of a functional glycolytic metabolon on mitochondria for metabolic efficiency. Nat. Metab..

[116] Moraes T.F., Reithmeier R.A.F. (2012). Membrane transport metabolons. Biochim. Biophys. Acta.

[117] Coelho R.G., Calaça I.C., Celestrini D.M., Correia-Carneiro A.H.P., Costa M.M., Zancan P., Sola-Penna M. (2015). Hexokinase and phosphofructokinase activity and intracellular distribution correlate with aggressiveness and invasiveness of human breast carcinoma. Oncotarget.

[118] Meira D.D., Marinho-Carvalho M.M., Teixeira C.A., Veiga V.F., Da Poian A.T., Holandino C., de Freitas M.S., Sola-Penna M. (2005). Clotrimazole decreases human breast cancer cells viability through alterations in cytoskeleton-associated glycolytic enzymes. Mol. Genet. Metab..

[119] El-Bacha T., de Freitas M.S., Sola-Penna M. (2003). Cellular distribution of phosphofructokinase activity and implications to metabolic regulation in human breast cancer. Mol. Genet. Metab..

[120] Zhan H., Pal D.S., Borleis J., Deng Y., Long Y., Janetopoulos C., Huang C.-H., Devreotes P.N. (2025). Self-organizing glycolytic waves tune cellular metabolic states and fuel cancer progression. Nat. Commun..

[121] Zhan H., Bhattacharya S., Cai H., Iglesias P.A., Huang C.-H., Devreotes P.N. (2020). An Excitable Ras/PI3K/ERK Signaling Network Controls Migration and Oncogenic Transformation in Epithelial Cells. Dev. Cell.

[122] Jeon M., Chauhan K.M., Szeto G.L., Kyoung M., An S. (2022). Subcellular regulation of glucose metabolism through multienzyme glucosome assemblies by EGF-ERK1/2 signaling pathways. J. Biol. Chem..

[123] Hansen H.L., Webb B.A. (2025). Functional requirements of the liver isoform of phosphofructokinase-1 in breast cancer cell migration. J. Cell Sci..

[124] Jeon M., Schmitt D.L., Kyoung M., An S. (2023). Size-Specific Modulation of a Multienzyme Glucosome Assembly during the Cell Cycle. ACS Bio Med. Chem. Au.

[125] Schmitt D.L., Dranchak P., Parajuli P., Blivis D., Voss T., Kohnhorst C.L., Kyoung M., Inglese J., An S. (2023). High-throughput screening identifies cell cycle-associated signaling cascades that regulate a multienzyme glucosome assembly in human cells. PLoS ONE.

[126] Fontana F., Giannitti G., Marchesi S., Limonta P. (2024). The PI3K/Akt Pathway and Glucose Metabolism: A Dangerous Liaison in Cancer. Int. J. Biol. Sci..

[127] Markham A. (2019). Alpelisib: First Global Approval. Drugs.

[128] Turner N.C., Oliveira M., Howell S.J., Dalenc F., Cortes J., Gomez Moreno H.L., Hu X., Jhaveri K., Krivorotko P., Loibl S. (2023). CAPItello-291 Study Group Capivasertib in Hormone Receptor-Positive Advanced Breast Cancer. N. Engl. J. Med..

[129] Pascual J., Turner N.C. (2019). Targeting the PI3-kinase pathway in triple-negative breast cancer. Ann. Oncol. Off. J. Eur. Soc. Med. Oncol..

[130] Juric D., Castel P., Griffith M., Griffith O.L., Won H.H., Ellis H., Ebbesen S.H., Ainscough B.J., Ramu A., Iyer G. (2015). Convergent loss of PTEN leads to clinical resistance to a PI(3)Kα inhibitor. Nature.

[131] Dent R., Oliveira M., Isakoff S.J., Im S.-A., Espié M., Blau S., Tan A.R., Saura C., Wongchenko M.J., Xu N. (2021). Final results of the double-blind placebo-controlled randomized phase 2 LOTUS trial of first-line ipatasertib plus paclitaxel for inoperable locally advanced/metastatic triple-negative breast cancer. Breast Cancer Res. Treat..

[132] Dent R.A., Kim S.-B., Oliveira M., Barrios C., O’Shaughnessy J., Isakoff S.J., Saji S., Freitas-Junior R., Philco M., Bondarenko I. (2024). Ipatasertib plus Paclitaxel for Patients with PIK3CA/AKT1/PTEN-Altered Locally Advanced Unresectable or Metastatic Triple-Negative Breast Cancer in the IPATunity130 Phase III Trial. Clin. Cancer Res. An. Off. J. Am. Assoc. Cancer Res..

[133] Guerra F.S., de Oliveira R.G., Fraga C.A.M., Mermelstein C.D.S., Fernandes P.D. (2017). ROCK inhibition with Fasudil induces beta-catenin nuclear translocation and inhibits cell migration of MDA-MB 231 human breast cancer cells. Sci. Rep..

[134] Lambert J.M.R., Gorzov P., Veprintsev D.B., Söderqvist M., Segerbäck D., Bergman J., Fersht A.R., Hainaut P., Wiman K.G., Bykov V.J.N. (2009). PRIMA-1 reactivates mutant p53 by covalent binding to the core domain. Cancer Cell.

[135] Park H., Shapiro G.I., Gao X., Mahipal A., Starr J., Furqan M., Singh P., Ahrorov A., Gandhi L., Ghosh A. (2022). Phase Ib study of eprenetapopt (APR-246) in combination with pembrolizumab in patients with advanced or metastatic solid tumors. ESMO Open.

[136] Bross P.F., Cohen M.H., Williams G.A., Pazdur R. (2002). FDA drug approval summaries: Fulvestrant. Oncologist.

[137] Verma S., Bartlett C.H., Schnell P., DeMichele A.M., Loi S., Ro J., Colleoni M., Iwata H., Harbeck N., Cristofanilli M. (2016). Palbociclib in Combination With Fulvestrant in Women With Hormone Receptor-Positive/HER2-Negative Advanced Metastatic Breast Cancer: Detailed Safety Analysis From a Multicenter, Randomized, Placebo-Controlled, Phase III Study (PALOMA-3). Oncologist.

[138] Turner N.C., Im S.-A., Saura C., Juric D., Loibl S., Kalinsky K., Schmid P., Loi S., Sunpaweravong P., Musolino A. (2024). Inavolisib-Based Therapy in PIK3CA-Mutated Advanced Breast Cancer. N. Engl. J. Med..

[139] Wu N., Zheng B., Shaywitz A., Dagon Y., Tower C., Bellinger G., Shen C.-H., Wen J., Asara J., McGraw T.E. (2013). AMPK-dependent degradation of TXNIP upon energy stress leads to enhanced glucose uptake via GLUT1. Mol. Cell.

[140] Wu Q., Ba-Alawi W., Deblois G., Cruickshank J., Duan S., Lima-Fernandes E., Haight J., Tonekaboni S.A.M., Fortier A.-M., Kuasne H. (2020). GLUT1 inhibition blocks growth of RB1-positive triple negative breast cancer. Nat. Commun..

[141] Chen Q., Meng Y.-Q., Xu X.-F., Gu J. (2017). Blockade of GLUT1 by WZB117 resensitizes breast cancer cells to adriamycin. Anti-Cancer Drugs.

[142] Zhao F., Ming J., Zhou Y., Fan L. (2016). Inhibition of Glut1 by WZB117 sensitizes radioresistant breast cancer cells to irradiation. Cancer Chemother. Pharmacol..

[143] Li Y.-L., Weng H.-C., Hsu J.-L., Lin S.-W., Guh J.-H., Hsu L.-C. (2019). The Combination of MK-2206 and WZB117 Exerts a Synergistic Cytotoxic Effect Against Breast Cancer Cells. Front. Pharmacol..

[144] Martel F., Guedes M., Keating E. (2016). Effect of polyphenols on glucose and lactate transport by breast cancer cells. Breast Cancer Res. Treat..

[145] Azevedo C., Correia-Branco A., Araújo J.R., Guimarães J.T., Keating E., Martel F. (2015). The chemopreventive effect of the dietary compound kaempferol on the MCF-7 human breast cancer cell line is dependent on inhibition of glucose cellular uptake. Nutr. Cancer.

[146] Temre M.K., Yadav S., Goel Y., Pandey S.K., Kumar A., Singh S.M. (2022). Glutor, a Glucose Transporter Inhibitor, Exerts Antineoplastic Action on Tumor Cells of Thymic Origin: Implication of Modulated Metabolism, Survival, Oxidative Stress, Mitochondrial Membrane Potential, pH Homeostasis, and Chemosensitivity. Front. Oncol..

[147] Banik S.M., Pedram K., Wisnovsky S., Ahn G., Riley N.M., Bertozzi C.R. (2020). Lysosome-targeting chimaeras for degradation of extracellular proteins. Nature.

[148] Liu Z., Qin Z., Liu Y., Xia X., He L., Chen N., Hu X., Peng X. (2023). Liquid—liquid phase separation: Roles and implications in future cancer treatment. Int. J. Biol. Sci..

[149] Bischof H., Cisarova K., Burgstaller S., Schwerer M., Absenger-Novak M., Jost P.J., Malli R., Graier W.F., Lukowski R. (2025). Targeting hexokinase 2 to induce breast cancer cell senescence. Br. J. Pharmacol..

[150] Li J., Pan J., Liu Y., Luo X., Yang C., Xiao W., Li Q., Yang L., Zhang X. (2022). 3-Bromopyruvic acid regulates glucose metabolism by targeting the c-Myc/TXNIP axis and induces mitochondria-mediated apoptosis in TNBC cells. Exp. Ther. Med..

[151] Wu Y., Sarkissyan M., Mcghee E., Lee S., Vadgama J.V. (2015). Combined inhibition of glycolysis and AMPK induces synergistic breast cancer cell killing. Breast Cancer Res. Treat..

[152] Feng X., Wang P., Liu Q., Zhang T., Mai B., Wang X. (2015). Glycolytic inhibitors 2-deoxyglucose and 3-bromopyruvate synergize with photodynamic therapy respectively to inhibit cell migration. J. Bioenerg. Biomembr..

[153] Zhong D., Xiong L., Liu T., Liu X., Liu X., Chen J., Sun S.-Y., Khuri F.R., Zong Y., Zhou Q. (2009). The glycolytic inhibitor 2-deoxyglucose activates multiple prosurvival pathways through IGF1R. J. Biol. Chem..

[154] Sang R., Fan R., Deng A., Gou J., Lin R., Zhao T., Hai Y., Song J., Liu Y., Qi B. (2023). Degradation of Hexokinase 2 Blocks Glycolysis and Induces GSDME-Dependent Pyroptosis to Amplify Immunogenic Cell Death for Breast Cancer Therapy. J. Med. Chem..

[155] Yi X., Qi M., Huang M., Zhou S., Xiong J. (2022). Honokiol Inhibits HIF-1α-Mediated Glycolysis to Halt Breast Cancer Growth. Front. Pharmacol..

[156] Spitz G.A., Furtado C.M., Sola-Penna M., Zancan P. (2009). Acetylsalicylic acid and salicylic acid decrease tumor cell viability and glucose metabolism modulating 6-phosphofructo-1-kinase structure and activity. Biochem. Pharmacol..

[157] Li W., Liu J., Jackson K., Shi R., Zhao Y. (2014). Sensitizing the therapeutic efficacy of taxol with shikonin in human breast cancer cells. PLoS ONE.

[158] Chen J., Xie J., Jiang Z., Wang B., Wang Y., Hu X. (2011). Shikonin and its analogs inhibit cancer cell glycolysis by targeting tumor pyruvate kinase-M2. Oncogene.

[159] Coelho R.G., Calaça I.d.C., Celestrini D.d.M., Correia A.H., Costa M.A.S.M., Sola-Penna M. (2011). Clotrimazole disrupts glycolysis in human breast cancer without affecting non-tumoral tissues. Mol. Genet. Metab..

[160] Motawi T.M.K., Sadik N.A.H., Fahim S.A., Shouman S.A. (2015). Combination of imatinib and clotrimazole enhances cell growth inhibition in T47D breast cancer cells. Chem.-Biol. Interact..

[161] Cao C., Lv C., ElFar A. (2026). Targeting phosphofructokinase in cancer: Integrating natural products for metabolic reprogramming and therapeutic innovation. Front. Pharmacol..

[162] Lunetti P., Di Giacomo M., Vergara D., De Domenico S., Maffia M., Zara V., Capobianco L., Ferramosca A. (2019). Metabolic reprogramming in breast cancer results in distinct mitochondrial bioenergetics between luminal and basal subtypes. FEBS J..

[163] Orobator O.N., Mertens R.T., Obisesan O.A., Awuah S.G. (2024). Energy and endoplasmic reticulum stress induction by gold(III) dithiocarbamate and 2-deoxyglucose synergistically trigger cell death in breast cancer. J. Biol. Chem..

[164] Lucantoni F., Dussmann H., Prehn J.H.M. (2018). Metabolic Targeting of Breast Cancer Cells With the 2-Deoxy-D-Glucose and the Mitochondrial Bioenergetics Inhibitor MDIVI-1. Front. Cell Dev. Biol..

[165] Guo X., Huang M., Zhang H., Chen Q., Hu Y., Meng Y., Wu C., Tu C., Liu Y., Li A. (2022). A pan-cancer analysis of thioredoxin-interacting protein as an immunological and prognostic biomarker. Cancer Cell Int..

[166] Stolearenco V., Levring T.B., Nielsen H.M., Lindahl L., Fredholm S., Kongsbak-Wismann M., Willerslev-Olsen A., Buus T.B., Nastasi C., Hu T. (2021). The Thioredoxin-Interacting Protein TXNIP Is a Putative Tumour Suppressor in Cutaneous T-Cell Lymphoma. Dermatology.

[167] Hu J., Feng L., Ren M., Zhao Y., Lu G., Lu X., Li Y., Wang X., Bu X., Wang S. (2022). Colorectal Cancer Cell Differentiation Is Dependent on the Repression of Aerobic Glycolysis by NDRG2-TXNIP Axis. Dig. Dis. Sci..

[168] Kim J.Y., Kim M.-J., Shin J.H., Baik K.-H., Kang Y., Sung Y., Kim S.H., Lee H.-J., Lee C., Shim J.-H. (2025). TXNIP Suppresses Lung Cancer Progression by Inhibiting TRAF6-Mediated NF-κB Activation and Autophagy. Immune Netw..

[169] Zhang Y., Yan Q., Gong L., Xu H., Liu B., Fang X., Yu D., Li L., Wei T., Wang Y. (2021). C-terminal truncated HBx initiates hepatocarcinogenesis by downregulating TXNIP and reprogramming glucose metabolism. Oncogene.

